# Supplementary figures and images for: Long-term outcomes of combination therapy with stereotactic body radiation therapy plus cryoablation using liquid nitrogen for stage I non-small cell lung cancer with tumors ≥2 cm
Source: PLoS One. 2025 Oct 8;20(10):e0332893. doi: 10.1371/journal.pone.0332893 (PMC12507226; doi:10.1371/journal.pone.0332893)

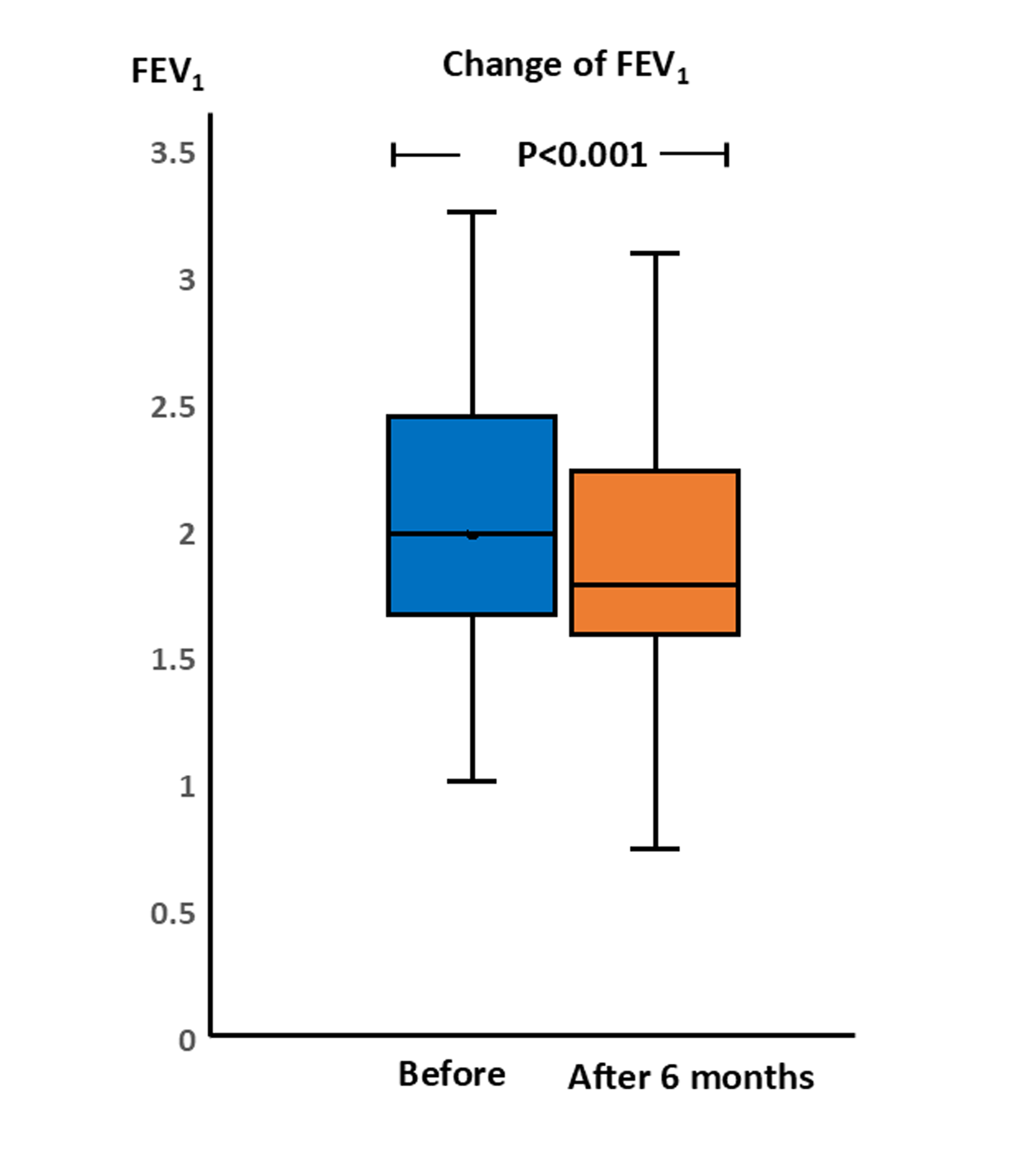

Supplement: S1 Fig — FEV1 significantly differs between the pre- and post-treatment (1.8 ± 0.6 vs. 1.7 ± 0.5 L, P < 0.001), with a mean post-/pre-FEV1 percentage of 95 ± 10% (median: 96; interquartile range: 90–100). FEV1, forced expiratory volume in 1 second. (TIF) [file pone.0332893.s001.tif]
