# Supplementary material for: Long-term outcomes of combination therapy with stereotactic body radiation therapy plus cryoablation using liquid nitrogen for stage I non-small cell lung cancer with tumors ≥2 cm
Source: PLoS One. 2025 Oct 8;20(10):e0332893. doi: 10.1371/journal.pone.0332893 (PMC12507226; doi:10.1371/journal.pone.0332893)
Supplement: S1 Table — (DOCX) [file pone.0332893.s002.docx]

Supplement Table 1. Characteristics of patients with local recurrence

No. Age/Sex Tumor size Histology Local recurrence Treatment Prognosis (months)

1 70/F 3.1 cm Sq 61 months None Dead with other disease (61)*

2 86/M 2.3 cm Ad 33 months None Dead with primary disease (41)

3 68/M 2.8 cm Sq 16 months Surgery Dead with primary disease (44)

4 58/M 2.6 cm Ad 13 months Chemo Dead with primary disease (29)

5 78/M 3.0 cm Sq 8 months Surgery Alive without disease (9)

F, female; M, male; Sq, squamous cell carcinoma; Ad: adenocarcinoma.

* Local recurrence found at autopsy for another disease.
