# Supplementary material for: Long-term outcomes of combination therapy with stereotactic body radiation therapy plus cryoablation using liquid nitrogen for stage I non-small cell lung cancer with tumors ≥2 cm
Source: PLoS One. 2025 Oct 8;20(10):e0332893. doi: 10.1371/journal.pone.0332893 (PMC12507226; doi:10.1371/journal.pone.0332893)
Supplement: S2 Table — (DOCX) [file pone.0332893.s003.docx]

Supplement Table 2. Characteristics of patients with pneumonitis

Pneumonitis

No. Age/Sex FEV_1_/FVC(%) Tumor size Onset Grade Prognosis (months)

1 80/F 79 3.1 cm 7 weeks 2 Dead with other disease (87)

2 79/F 55 2.2 cm 9 weeks 3 Dead with other disease (61)

3 82/F 71 2.7 cm 11 weeks 2 Dead with other disease (61)

4 80/M 58 4.0 cm 12 weeks 2 Dead with primary disease(57)

5 77/F 71 2.4 cm 15 weeks 2 Alive without disease (72)

FEV_1_/FVC, forced expiratory volume in 1 second / forced vital capacity;

Onset, onset time of pneumonitis after stereotactic radiation therapy;

F, female; M, male.
