# Supplementary material for: Long-term outcomes of combination therapy with stereotactic body radiation therapy plus cryoablation using liquid nitrogen for stage I non-small cell lung cancer with tumors ≥2 cm
Source: PLoS One. 2025 Oct 8;20(10):e0332893. doi: 10.1371/journal.pone.0332893 (PMC12507226; doi:10.1371/journal.pone.0332893)
Supplement: S3 Table — (DOCX) [file pone.0332893.s004.docx]

Supplement Table 3. Cox proportional hazard regression analysis

for local recurrence

Univariate analysis

Variables HR (95% CI) P-value

Tumor size 1.5 (0.3–7.0) 0.62

Tumor stage 2.0 (0.6–6.9) 0.27

Tumor histology (Ad vs. Sq) 3.1 (0.5–18.3) 0.22

SUV on PET 1.1 (0.9–1.3) 0.53

BED 1.0 (0.9–1.1) 0.63

HR, hazard ratio; CI, confidence interval;

Ad, adenocarcinoma; Sq, squamous cell carcinoma;

SUV, standardized uptake value; PET, positron emission tomography;

BED, biological effective dose.
