# Supplementary material for: Long-term outcomes of combination therapy with stereotactic body radiation therapy plus cryoablation using liquid nitrogen for stage I non-small cell lung cancer with tumors ≥2 cm
Source: PLoS One. 2025 Oct 8;20(10):e0332893. doi: 10.1371/journal.pone.0332893 (PMC12507226; doi:10.1371/journal.pone.0332893)
Supplement: Attachment — Submitted filename: RevGeneralComments.docx [file pone.0332893.s005.docx]

**Major Comments**

The manuscript presents a well-designed retrospective cohort study on the long-term outcomes of combined SBRT and cryoablation for cT1-2N0M0 NSCLC with tumor size ≥2 cm. The median follow-up of 73 months and focus on a clinically relevant population – patients ineligible or unwilling to undergo surgery – enhance the significance of this work. The results are clearly reported and show promising local control and overall survival outcomes.

However, several areas require clarification, elaboration, and improvement to strengthen the manuscript's scientific rigor and clinical impact.

1. Novelty and Contextualization

The rationale for combining SBRT and cryoablation is interesting but not fully contextualized in relation to prior combination therapy reports (if any exist). A more explicit explanation of how this study is novel compared to previous SBRT or ablation-alone studies is warranted.

2. Patient Selection

- The study mixes patients who were medically inoperable and those who preferred non-surgical treatment. This creates heterogeneity in the cohort. Please discuss how this choice affects generalizability and whether outcomes differ between these two groups.
- Inclusion of two patients with stage II NSCLC (T2bN0M0) should be acknowledged and justified more explicitly or excluded for consistency with the stated focus on Stage I disease.

3. SBRT Technique Clarification

- The SBRT section lacks clarity on how tumor motion was handled quantitatively. Was 4D-CT used? Are the three free-breathing scans equivalent to a poor-man's ITV?
- The use of a 5 mm margin to define PTV may be standard, but justification or comparison with motion management strategies (e.g., gating, abdominal compression) would be helpful.

4. Statistical Analysis

- Multivariate analyses were conducted despite small event numbers (e.g., only 6 pneumonia events). Please clarify how overfitting or type II errors were mitigated, or consider simplifying the analysis or using Firth’s correction where applicable.
- The use of Microsoft Excel 10 for statistical analysis may not inspire confidence in reproducibility. Consider using a more robust statistical platform (e.g., R, SPSS, or Stata) or at least justify the use of Excel.

5. Reported events

- There is no grading of pneumothorax severity. Consider including this to align with CTCAE standards.
- The decrease in FEV1 is statistically significant but arguably not clinically meaningful. This distinction should be emphasized.

While the manuscript aims to compare cryoablation and SBRT for early-stage NSCLC, it currently lacks sufficient detail regarding the cryoablation technique. For the study to be scientifically rigorous and clinically informative, it is important that the authors provide a comprehensive description of the cryoablation procedure. This should include, but not be limited to probe placement strategy, imaging modality for guidance, lesion size eligibility, and criteria for defining adequate ablation margins. Without this information, readers are unable to fully assess the comparability of the two treatment modalities, and the reproducibility of the cryoablation approach is compromised. I recommend that the authors include this information in the revised manuscript.

6. Lack of details on the Cryoablation Technique

There is no information on imaging guidance used, lesion size limitation, or margin assessment. Without understanding how cryoablation was applied, it’s difficult for readers to assess if the outcomes were influenced by technical variability, operator experience, or treatment adequacy.

_____________________________________________________________________________

**Minor Comments**

1. Language and Style

- The text is mostly clear but would benefit from professional editing for grammar, flow, and tense consistency (e.g., “conducted the cryoablation” → “cryoablation was performed”).
- “Study treatment” is vague and repetitive. Consider replacing with “combination therapy” or “SBRT-cryoablation regimen” for clarity.

2. Formatting

- Ensure consistent use of abbreviations (e.g., CTCAE, SUV, BED) and define them on first use.
- The phrase “on the other hand” is overused in the Introduction. Rephrasing or restructuring is recommended for better logical flow.

____________________________________________________________________________

**Decision**

This manuscript addresses an important and clinically relevant topic by exploring a relatively under-reported combination therapy. While the study has the potential to contribute meaningfully to the field of lung cancer treatment, it currently lacks sufficient detail in several key areas. Therefore, I do not recommend acceptance in its current form. A major revision is required to strengthen the scientific content and provide a more comprehensive and informative discussion that highlights the therapeutic value and clinical implications of this approach.
