# Supplementary material for: Long-term outcomes of combination therapy with stereotactic body radiation therapy plus cryoablation using liquid nitrogen for stage I non-small cell lung cancer with tumors ≥2 cm
Source: PLoS One. 2025 Oct 8;20(10):e0332893. doi: 10.1371/journal.pone.0332893 (PMC12507226; doi:10.1371/journal.pone.0332893)
Supplement: Attachment — Submitted filename: renamed_22856_minorcomments.docx [file pone.0332893.s006.docx]

Long-term outcomes of combination therapy with stereotactic body radiation therapy and cryoablation for T1-2N0M0 non-small cell lung cancer with tumor size ≥2 cm

Hiroaki Nomori, MD^1^; Yue Cong MD^2^; Hiroyoshi Iguchi, MD^3^; Kenichi Kashihara, MD^3^; Ryuichi Wada, MD^4^; Tsutomu Saito, MD^5^

Departments of Thoracic Surgery of Kashiwa Kousei General Hospital^1^,

Department of Thoracic Surgery, Tokyo University Hospital^2^,

Departments of Radiology^3^, and Pathology^4^ of Kashiwa Kousei General Hospital,

Department of Radiology, Sonodakai Radiation Clinic^5^.

Corresponding author: Hiroaki Nomori, MD, Department of General Thoracic Surgery, Kashiwa Kousei General Hospital. 617 Shikoda, Kashiwa city, 277-8551, Chiba, Japan.

Tel: +81-471-45-1111.

E-mail: [hnomori@qk9.so-net.ne.jp](mailto:hnomori@qk9.so-net.ne.jp)

Registration information of the study

The study was approved by ethical committees of the two institutes, i.e., No.1 and No.2, both of which are also registered to UMIN University Hospital Medical Information (UMIN) clinical trial.

Registration date and number:

No. 1, 2015/8/19. 15-011 in ethical committee. UMIN18573.

No.2, 2019/4/11. 19-02 in ethical committee. UMIN 37215.

The name of the study in UMIN registration:

No.1: Prospective study of safety and efficacy of additional cryoablation after radiation therapy for malignant lung tumors.

No.2: Radiation therapy followed by cryoablation for malignant lung tumors.

The study period:

No.1: from 2015 to 2018.

No.2: from 2019 until now.

**Abstract**

**Objectives**: While the radiation or ablation therapy has been conducted for T1N0M0 lung cancer, most of their survival have been examined with 3-year, which is not long enough. In fact, the 5-year survival of stereotactic body radiation for stage I lung cancer was reportedly decreased to 40%. This study aimed to assess 5-year treatment results with combination therapy of stereotactic body radiation and cryoablation for T1-2N0M0 non-small cell lung cancer with tumor size ≥2 cm with 73 months of median follow-up.

**Methods**: This retrospective observational study included 66 patients with T1-2N0M0 non-small cell lung cancer ≥2 cm who underwent stereotactic body radiation therapy followed by cryoablation between 2015 and 2023. All tumors were pathologically diagnosed before treatment. Patients received stereotactic body radiation therapy (40–60 Gy / 4–10 fractions) with a median biological effective dose of 100 Gy, followed by cryoablation using liquid nitrogen within 3 weeks. Study outcomes included local control, survival, and adverse events. Median follow-up duration was 73 months (range: 3–111).

**Results:** The mean tumor diameter was 2.7 ± 0.7 cm (range: 2.0–5.4). Five patients (8%) experienced local recurrence at 8–61 months after treatment. The 5-year rates of local control and overall survival were 93% and 75%, respectively. There were no cases of treatment-related mortality. The most frequent complication was post-cryoablation pneumothorax (38%), with the median drainage period being 2 days. Six (9%) patients experienced pneumonitis, including radiation pneumonitis (n=5) and bronchopneumonia (n=1), of which severity was grade 2 in five and 3 in one; nonetheless, all these patients recovered without any sequelae.

**Conclusion****:** Stereotactic body radiation therapy followed by cryoablation is a feasible treatment for T1-2N0M0 non-small cell lung cancer with tumor size ≥2 cm, with improved survival and acceptable rates of adverse events, compared to stereotactic body radiation alone.

**Keywords:** stereotactic body radiation therapy, cryoablation, liquid nitrogen, combination therapy, non-small cell lung cancer, T1-2N0M0, minimal invasive treatment

**Abbreviations and Acronyms**

BED	= biological effective dose

CI	= 95% confidence interval

CTCAE= Common Terminology Criteria for Adverse Events

CT	= computed tomography

DSS	=disease specific survival

FDG-PET=fluorodeoxyglucose-positron emission tomography

FEV_1_	= forced expiratory volume in 1 s

FVC	= forced vital capacity

IQR	= interquartile range

LC	=local control

NSCLC	= non-small cell lung cancer

OS	= overall survival

RFA	= radiofrequency ablation

RFS	= recurrence-free survival

SBRT	= stereotactic body radiation therapy

SUV	= standardized uptake value

TNM	= stage with Tumor, Node, and Metastasis.

**Introduction**

Stereotactic body radiotherapy (SBRT) has become the first choice of treatment for inoperable patients with clinical stage I non-small cell lung cancer (NSCLC) [1-7]. However, there are significant differences between the local control (LC) and overall survival (OS) rates in these reports (≥90% vs. 43–83% respectively). In addition, a meta-analysis found that surgery led to a significantly longer OS than SBRT in patients with stage I NSCLC [8]. While the low OS rate after SBRT could be attributed to a high comorbidity in inoperable patients, it could also result from residual tumors within scar tissue that developed after SBRT, which are usually hard to detect on computed tomography (CT) and could metastasize, and thus would decrease the OS after SBRT. Additionally, most studies on SBRT reported the 3-year OS rate [1-5], which is too short for assessing treatment outcomes in patients with stage I NSCLC. The study conducted by Timmerman et al. on SBRT for stage I NSCLC showed 56% of 3-year study [2], which was decreased to 40% in their 5-year assessment [6]. Shintani et al. reported that local recurrence occurred even at >5 years after SBRT [9].

On the other hand, ablation therapies such as cryoablation or radiofrequency ablation (RFA) have been also reported as alternative therapies for stage I NSCLC [10-13]. The 3-year LC and OS rates after cryoablation for stage I NSCLC have been reported to be 90% and 95%, respectively, with no significant difference unlike in SBRT [10]. However, the LC rates after both cryoablation and RFA are lower for NSCLCs with tumor size ≥2 cm than those <2 cm [10,11], which is consistent with the findings regarding SBRT [9].

Combination therapy with SBRT, cryoablation, and RFA for stage I NSCLC with tumor size ≥2 cm may allow improving LC and survival; however, this remains to be studied. Although RFA reportedly increases the incidence of pneumonia in patients with NSCLC who have previously undergone external beam radiotherapy [12], cryoablation shows no difference in the incidence of adverse events between patients with and without interstitial pneumonia [13]. Therefore, cryoablation is more acceptable than RFA for combination with SBRT.

In combination therapy with SBRT and cryoablation, SBRT should be performed first, because tumor radiosensitivity is dependent on intracellular oxygen concentration [14]. Because cryoablation decreases tumor vascularity, this would decrease tumor radiosensitivity. Additionally, scarring around the tumor usually occurs immediately after cryoablation, which impedes SBRT planning. In contrast, SBRT usually does not make the scar within 1 month. Therefore, this study conducted the cryoablation 2 to 3 weeks after SBRT for 66 patients with cT1-2N0M0 NSCLC with tumor size ≥2 cm, and evaluated the long-term outcomes with a median follow-up period of 73 months.

**Materials and Methods**

**Study design and setting**

This descriptive retrospective cohort study adhered to the Strengthening the Reporting of Observational Studies in Epidemiology guidelines [15] and was conducted in accordance with the [Declaration of Helsinki](http://www.wma.net/en/30publications/10policies/b3/). The study was approved in 2015 (15-011) and 2020 (20-03) by the two institutional ethics committees.

**Eligibility**

All tumors were pathologically diagnosed as NSCLC by using needle or bronchoscopic biopsy before treatment. Tumor stage was confirmed using 18F-fluorodeoxyglucose-positron emission tomography (FDG-PET). The inclusion criteria were as follows: (1) clinical T1-2N0M0 peripheral-type NSCLC with tumor size ≥2 cm based on the 8^th^ edition of TNM classification by the International Association for the Study of Lung Cancer [16]; (2) Eastern Cooperative Oncology Group performance status 0–2; (3) patients ineligible for surgery due to comorbidities, low FEV_1_, or old age and (4) patients who preferred the study treatment to surgery. The exclusion criteria were as follows: (1) active interstitial pneumonia or previous treatment for interstitial pneumonia; and (2) tumors located outside the reach of the cryoprobe. All patients provided written informed consent after receiving extensive explanation regarding the risks and benefits of treatment.

**SBRT**

SBRT was administered using an Electa Versa HD (Electa Co. Ltd., Tokyo, Japan). Respiratory motion was managed using three sets of conventional free-breathing CT simulation images, with or without the use of a breath limitation device.

Internal tumor volume encompassing the full range of tumor motion was delineated using the second and the third images of the clinical target volume of the first image. A planning target volume expansion of 5 mm was applied to the internal tumor volume. Fractionation schemas were determined by radiation oncologists to optimize the planning target volume coverage and meet organ-risk dose constraints based on Radiation Therapy Oncology Group (RTOG) 0236, 0813, and 0915 trials. Each fractionation schedule contained a biological effective dose (BED) of at least 80 Gy (α/β=10). Treatment courses were administered using a consecutive-day, one-daily fractionation schedule.

**Cryoablation**

At 2–3 weeks after SBRT, cryoablation was performed using liquid nitrogen (IceSense 3; IceCure Medical Ltd., Caesarea, Israel) and a cryoprobe 3.4 mm in diameter. To guide the cryoprobe to the tumor, a guide needle kit (Daimon coaxial system; Silux Co., Kawaguchi, Japan) was used, comprising a 21-G guide needle and an 11-G stainless-steel coaxial system, which consisted of an inner guide sheath and outer sheath (**Fig. 1**).

Cryoablation was performed under local anesthesia and sedation, usually with one probe and three cycles as follows: 5-min freeze and 8-min thaw; 8-min freeze and 10-min thaw; and 8-min freeze and 4-min active thaw, yielding a total of treatment duration of 43 min.

**Follow-up**

Follow-up using CT was conducted every 3 or 4 months until 3 years after treatment, with a minimum 6-month interval being subsequently applied. In a case of suspected local recurrence on CT images, needle aspiration biopsy was performed. The duration of LC and survival were calculated from treatment initiation with SBRT. Follow-up data were collected from chart abstractions on September 5^th^ in 2024.

**Safety assessment**

Posttreatment adverse events were graded based on the Common Terminology Criteria for Adverse Events (CTCAE version 4.03) [17]. Pneumonitis was defined with CT images and the presence of related symptoms. Pneumothorax after cryoablation was defined by the requirement of thoracic drainage.

**Evaluation of preserved pulmonary function**

Forced expiratory volume in 1 s (FEV_1_) and forced vital capacity (FVC) were measured before treatment and at 6 months after treatment. The percentage of preserved pulmonary function was calculated as follows: post-FEV_1_ /pre-FEV_1_× 100 (%).

**Variables**

Comorbidities were assessed using the Charlson comorbidity index [18]. Accumulation on FDG-PET was evaluated using the standardized uptake value (SUV). Radiation dose was assessed using the BED. V20 was used as an indicator of adverse effects of SBRT.

**Local recurrence**

Local recurrence was indicated when needle biopsy showed malignancy or the primary site apparently increased. Other recurrences, including metastases to the hilar/mediastinal lymph nodes or lungs outside the primary site, were defined as distant metastases.

**Study outcomes**

The primary outcomes were LC, OS, recurrence-free survival (RFS), disease-specific survival (DSS), and adverse events. The secondary outcome was a change in pulmonary function.

**Statistical analysis**

Kaplan-Meier method was used to evaluate LC and survival. Univariate cox proportional hazards regression analysis was used to assess variables related to LC and survival. Univariate logistic regression analysis was used to assess variables associated with pneumonia. Variables with P≤0.15 in the univariate analysis were entered into the multivariate analysis. Wilcoxon signed-rank test was used to compare FEV_1_ values before and after the treatment. Statistical significance was set at P<0.05. All statistical analyses were performed using Microsoft Excel version 10 (Redmond, WA, USA).

**Results**

Between 2015 and 2023, 333 patients with cT1-2N0M0 non-small cell lung cancer with tumor size ≥2 cm underwent local treatments, including surgery, SBRT, and cryoablation (**Fig. 2**). Among 80 patients who underwent cryoablation, 66 patients, including 2 patients with T2bN0M0 (Stage II), were treated by SBRT followed by cryoablation (**Table 1**). The reasons for selecting the study treatment included Charlson comorbidity index ≥3 (n=26), FEV_1_/FVC < 70% (n=36), and age ≥ 80 years (n=23). On the other hand, 40 (61%) patients requested the study treatment rather than surgery despite having a comorbidity index <3. The FDG-PET was conducted for 62 of the 66 patients (94%).

**Table 2** shows the treatment details. SBRT was administered with median fractions of 5 (interquartile range (IQR), 5–5), a median total dose of 50 Gy (IQR, 44–50), and a median BED of 100 Gy (IQR, 82–100). Cryoablation was conducted with a median number of 1 probe (IQR, 1–1) and 3 cycles (IQR, 3–4).

None of the patients were lost to follow-up. The median follow-up period was 73 months (range, 3–111; IQR, 42–88). Both the 3- and 5-year LC rates were 93% (95% confidence interval [CI], 87%–100%) (**Fig. 3**). Local recurrence was diagnosed in 5 (8%) patients between 8 and 61 months after the treatment (**Table 3**).

The 3- and 5-year OS rates were 87% (CI, 79%–96%) and 75% (CI, 64%–86%), respectively (**Fig. 4)**. During the follow-up period, 32 (48%) patients were dead, with 24 of them dying of other diseases.

The 3- and 5-year RFS rates were 74% (CI, 63%–84%) and 60% (CI, 48%–73%), respectively (**Fig. 5**). Fifteen patients (23%) experienced distant metastases between 3 and 60 months after the treatment.

The 3- and 5-year DSS rates were 97% (CI, 92%–100%) and 93% (CI, 86%–100%), respectively (**Fig. 6)**.

None of the patients experienced treatment-related mortality. Pneumothorax occurred in 25 (38%) patients after cryoablation, with the median chest tube drainage period being 2 days (IQR, 1–4). Median duration of hospital stay after cryoablation was 5 days (IQR, 4–5). Six (9%) patients developed pneumonitis with grade 2 (n=5) and grade 3 (n=1) on CTCAE criteria between 7 and 15 weeks after the treatment (**Table 4**); among them, five had radiation pneumonitis and one had bronchopneumonia. All of them recovered without any sequelae and survived between 36 and 86 months after the treatment. There were no cases of late-onset adverse events associated with SBRT, such as interstitial pneumonia, rib fracture, or pleural effusion.

Univariate cox regression analysis for LC revealed no correlations with tumor size, tumor stage, tumor histology (adenocarcinoma vs. squamous cell carcinoma), and SUV on PET (p=0.20 – 0.95) (**Table 5**).

Univariate cox regression analysis for OS revealed correlations (p≤0.15) with tumor stage (p=0.01), SUV on PET (p=0.02), and tumor histology (p=0.09); however, these correlations were not significant in the multivariate analysis (p=0.09–0.63) **(Table 6)**.

Univariate logistic regression analysis for pneumonia showed correlations (p≤0.15) with age (p=0.15), tumor size (p=0.11), and V20 (p=0.14); however, these correlations were not significant in the multivariate analysis (p=0.18–0.51) **(Table 7)**.

Pulmonary function tests were performed both before and 6 months after treatment in 61 (92%) 66 patients. There was a significant decrease in FEV_1_ between pretreatment and 6-month posttreatment (1.8 ± 0.6 vs. 1.7 ± 0.6 L, p<0.001), of which the mean post- /pre-FEV_1_ percentage was 96 ± 9% (median, 97; IQR, 92–102) (**Fig. 7**).

**Comment**

This study highlights the following points: (1) 5-year OS after SBRT followed by cryoablation was better than that reported in studies on SBRT alone; (2) the incidence rate of pneumonia after the combination therapy was similar to that after SBRT alone; (3) the decrease in pulmonary function was similar to that after SBRT alone; and (4) while pneumothorax after cryoablation was observed in 38% of the patients, the median drainage period was just 2 days.

Most studies on SBRT alone reported that the 3-year LC rate was >90% [1-7], with our findings indicating that both the 3- and 5- year LC rates were 93%. However, the high LC rates after SBRT, including those in the present study, can be misleading because of residual tumors in the large scars formed after SBRT, which could cause distant metastases. Therefore, treatment outcomes after SBRT should be evaluated based on survival rates.

In this study, the 3-year OS rates was 87%, which is equal to or higher than that reported in studies on SBRT alone (43%–83%) [1-5]. However, a 3-year duration is too short to estimate OS of patients with stage I NSCLC, which should be ≥5 years. Timmerman and Shibamoto reported the 5-year OS rates after SBRT alone to be 40% and 52%, respectively [6,7]. Contrastingly, the 5-year OS rate in our study was 75%, which suggests that SBRT followed by cryoablation could control primary tumors and prevent distant recurrences compared to SBRT alone.

Surgery is the standard therapy for stage I-II NSCLC. A nationwide lung cancer registry study of > 11,000 surgical cases reported that the 5-year OS rates for stages IA, IB, and IIA were 82, 67, and 54%, respectively [19], which is comparable to the 5-year OS in this study (75%).

Before the start of this study, the most significant concern was that cryoablation could increase the incidence of radiation pneumonitis caused by SBRT. The study showed that the pneumonitis higher than grade 2 was seen in six (9%) patients, which is not higher than its incidence after SBRT alone (7%–13 %) [4,5,7,20]. This suggests that cryoablation does not cause further inflammation after SBRT.

In this study, the pulmonary function at 6 months after the treatment was 96% of the pretreatment value, which is similar to the values after SBRT alone (92%–96%) [21-23]. Taken together, cryoablation after SBRT did not decrease pulmonary function compared to the SBRT alone.

Pneumothorax is known to be associated with cryoablation or RFA, of its incidence in the present study being 38%. However, the median drainage period was only 2 days, which was not a severe event.

This study has the following limitations: (1) the retrospective descriptive design and the lack of a control group impeded establishment of a causal inference between the treatment and outcome; and (2) a biopsy of the primary tumor was not routinely conducted at the time of distant metastases; therefore, LC could not be precisely estimated.

**Conclusion**

SBRT followed by cryoablation is a feasible treatment option for T1-2N0M0 non-small cell lung cancer with tumor size ≥2 cm, with improved survival and acceptable rates of adverse events, compared to stereotactic body radiation alone.

**References**

1. Nagata Y, Takayama K, Matsuo Y, Norihisa Y, Mizowaki T, Sakamoto T, et al. [Clinical outcomes of a phase I/II study of 48 Gy of stereotactic body radiotherapy in 4 fractions for primary lung cancer using a stereotactic body frame.](https://pubmed.ncbi.nlm.nih.gov/16169670/)　 Int J Radiat Oncol Biol Phys. 2005 Dec 1;63(5):1427-31. doi: 10.1016/j.ijrobp.2005.05.034. Epub 2005 Sep 19. PMID: 16169670.
2. Timmerman R, Paulus R, Galvin J, Michalski J, Straube W, Bradley J, et al. [Stereotactic body radiation therapy for inoperable early-stage lung cancer.](https://pubmed.ncbi.nlm.nih.gov/20233825/) JAMA. 2010 Mar 17;303(11):1070-6. doi:10.1001/jama.2010.261. PMID: 20233825.
3. Zimmermann FB, Geinitz H, Schill S, Thamm R, Nieder C, Schratzenstaller U, et al. [Stereotactic hypofractionated radiotherapy in stage I (T1-2 N0 M0) non-small-cell lung cancer (NSCLC).](https://pubmed.ncbi.nlm.nih.gov/16982542/) Acta Oncol. 2006;45(7):796-801. doi: 10.1080/02841860600913210. PMID: 16982542.
4. Fakiris AJ, McGarry RC, Yiannoutsos CT, Papiez L, Williams M, Henderson MA, et al. [Stereotactic body radiation therapy for early-stage non-small-cell lung carcinoma: four-year results of a prospective phase II study.](https://pubmed.ncbi.nlm.nih.gov/19251380/) Int J Radiat Oncol Biol Phys. 2009 Nov 1;75(3):677-82. doi: 10.1016/j.ijrobp.2008.11.042. Epub 2009 Feb 27. PMID: 19251380.
5. Nagata Y, Hiraoka M, Shibata T, Onishi H, Kokubo M, Karasawa K, et al. [Prospective Trial of Stereotactic Body Radiation Therapy for Both Operable and Inoperable T1N0M0 Non-Small Cell Lung Cancer: Japan Clinical Oncology Group Study JCOG0403.](https://pubmed.ncbi.nlm.nih.gov/26581137/) Int J Radiat Oncol Biol Phys. 2015 Dec 1;93(5):989-96. doi: 10.1016/j.ijrobp.2015.07.2278. Epub 2015 Nov 11. PMID: 26581137.
6. Timmerman RD, Hu C, Michalski JM, Bradley JC, Galvin J, Johnstone DW, et al. [Long-term results of stereotactic body radiation therapy in medically inoperable stage I non-small cell lung cancer.](https://pubmed.ncbi.nlm.nih.gov/29852036/) JAMA Oncol. 2018 Sep 1;4(9):1287-1288. doi: 10.1001/jamaoncol.2018.1258. PMID: 29852036.
7. Shibamoto Y, Hashizume C, Baba F, Ayakawa S, Miyakawa A, Murai T, et al. [Stereotactic body radiotherapy using a radiobiology-based regimen for stage I non-small-cell lung cancer: five-year mature results.](https://pubmed.ncbi.nlm.nih.gov/26001145/) J Thorac Oncol. 2015 Jun;10(6):960-4. doi: 10.1097/JTO.0000000000000525. PMID: 26001145.
8. Cao C, Wang D, Chung C, Tian D, Rimner A, Huang J, et al. [A systematic review and meta-analysis of stereotactic body radiation therapy versus surgery for patients with non-small cell lung cancer.](https://pubmed.ncbi.nlm.nih.gov/30482524/) Thorac Cardiovasc Surg. 2019 Jan;157(1):362-373.e8. doi: 10.1016/j.jtcvs.2018.08.075. Epub 2018 Sep 15. PMID: 30482524.
9. Shintani T, Matsuo Y, Iizuka Y, Mitsuyoshi T, Mizowaki T. [A retrospective long-term follow-up study of stereotactic body radiation therapy for non-small cell lung cancer from a single institution: Incidence of late local recurrence.](https://pubmed.ncbi.nlm.nih.gov/29722662/) Int J Radiat Oncol Biol Phys. 2018 Apr 1;100(5):1228-1236. doi: 10.1016/j.ijrobp.2018.01.050. Epub 2018 Jan 31. PMID: 29722662.
10. Nomori H, Yamazaki I, Shiraishi A, Adachi T, Kanno M. [Cryoablation for T1N0M0 non-small cell lung cancer using liquid nitrogen.](https://pubmed.ncbi.nlm.nih.gov/33152627/) Eur J Radiol. 2020 Dec,133:109334. doi: 10.1016/j.ejrad.2020.109334. Epub 2020 Oct 22. PMID: 33152627.
11. Huang BY, Li XM, Song XY, Zhou JJ, Shao Z, Yu ZQ, et al. [Long-term results of CT-guided percutaneous radiofrequency ablation of inoperable patients with stage Ia non-small cell lung cancer: A retrospective cohort study.](https://pubmed.ncbi.nlm.nih.gov/29555533/) Int J Surg. 2018 May;53:143-150. doi: 10.1016/j.ijsu.2018.03.034. Epub 2018 Mar 16. PMID: 29555533.
12. Kashima M, Yamakado K, Takaki H, Kodama H, Yamada T, Uraki J, et al. [Complications after 1000 lung radiofrequency ablation sessions in 420 patients: a single center's experiences.](https://pubmed.ncbi.nlm.nih.gov/21940529/) AJR Am J Roentgenol. 2011 Oct;197(4): W576-80. doi: 10.2214/AJR.11.6408. PMID: 21940529.
13. Sänger JA, Graur A, Tahir I, Price MC, Keane FK, Lanuti M, et al. [Outcomes following cryoablation of stage IA non-small cell lung cancer in patients with](https://pubmed.ncbi.nlm.nih.gov/37172368/)

[and without interstitial lung disease: A retrospective single-center cohort study.](https://pubmed.ncbi.nlm.nih.gov/37172368/) Lung Cancer. 2023 Jul; 181:107231. doi: 10.1016/j.lungcan.2023. 107231. Epub 2023 May 5. PMID: 37172368.

14. Jordan BF, Sonveaux P, Feron O, Grégoire V, Beghein N, Dessy C et al. [Nitric oxide as a radiosensitizer: evidence for an intrinsic role in addition to its effect on oxygen delivery and consumption.](https://pubmed.ncbi.nlm.nih.gov/14999787/) Int J Cancer. 2004 May 1;109(5):768-73. doi: 10.1002/ijc.20046.PMID: 14999787.
15. Vandenvbroucke JP, von Elm E, Altman DG, Gøtzsche PC, Mulrow CD, Pocock SJ, et al. Strengthening the Reporting of Observational Studies in Epidemiology (STROBE): Explanation and Elaboration. Epidemiology 2007; 18:805-835. doi: 10.1016/j.ijsu.2014.07.014. Epub 2014 Jul 18. PMID: 25046751.
16. Chansky K, Detterbeck FC, Nicholson AG, [Rusch VW](https://www.ncbi.nlm.nih.gov/pubmed/?term=Rusch%20VW%5BAuthor%5D&cauthor=true&cauthor_uid=28461257), [Vallières E](https://www.ncbi.nlm.nih.gov/pubmed/?term=Valli%C3%A8res%20E%5BAuthor%5D&cauthor=true&cauthor_uid=28461257), [Groome P](https://www.ncbi.nlm.nih.gov/pubmed/?term=Groome%20P%5BAuthor%5D&cauthor=true&cauthor_uid=28461257), et al. IASLC Staging and Prognostic Factors Committee, Advisory Boards, and Participating Institutions. [The IASLC Lung Cancer Staging Project: External Validation of the Revision of the TNM Stage Groupings in the Eighth Edition of the TNM Classification of Lung Cancer.](https://www.ncbi.nlm.nih.gov/pubmed/28461257) J Thorac Oncol. 2017; 12:1109-1121. doi: 10.1016/j.jtho.2017.04.011. Epub 2017 Apr 28. PMID: 28461257.
17. Trotti A, Colevas AD, Setser A, Rusch V, Jaques D, Budach V, et al. [CTCAE v3.0: development of a comprehensive grading system for the adverse effects of cancer treatment.](https://pubmed.ncbi.nlm.nih.gov/12903007/) Semin Radiat Oncol. 2003 Jul;13(3):176-81. doi: 10.1016/S1053-4296(03)00031-6. PMID: 12903007.
18. Charlson ME, Pompei P, Ales KL, MacKenzie CR. A new method of classifying prognostic comorbidity in longitudinal studies: Development and validation. J Chronic Dis 1987; 40: 373-83. doi: [10.1016/0021-9681(87)90171-8](https://doi.org/10.1016/0021-9681(87)90171-8). PMID: **3558716.**
19. Sawabata N, Miyaoka E, Asamura H, Nakanishi Y, Eguchi K, Mori M, et al. [Japanese lung cancer registry study of 11,663 surgical cases in 2004: demographic and prognosis changes over decade.](https://pubmed.ncbi.nlm.nih.gov/21610521/); Japanese Joint Committee for Lung Cancer Registration. J Thorac Oncol. 2011 Jul;6(7):1229-35. doi: 10.1097/JTO.0b013e318219aae2. PMID: 21610521.
20. Shinde A, Li R, Kim J, Salgia R, Hurria A, Amini A. [Stereotactic body radiation therapy (SBRT) for early-stage lung cancer in the elderly.](https://pubmed.ncbi.nlm.nih.gov/30286944/) Semin Oncol. 2018 Aug;45(4):210-219. doi: 10.1053/j.seminoncol.2018.06.002. Epub 2018 Jul 1. PMID: 30286944
21. [Guckenberger](https://pubmed.ncbi.nlm.nih.gov/?sort=date&term=Guckenberger+M&cauthor_id=23154077) M,  [Klement](https://pubmed.ncbi.nlm.nih.gov/?sort=date&term=Klement+RJ&cauthor_id=23154077) RJ, [Kestin](https://pubmed.ncbi.nlm.nih.gov/?sort=date&term=Kestin+LL&cauthor_id=23154077) LL,  [Hope](https://pubmed.ncbi.nlm.nih.gov/?sort=date&term=Hope+AJ&cauthor_id=23154077) AJ, [Belderbos](https://pubmed.ncbi.nlm.nih.gov/?sort=date&term=Belderbos+J&cauthor_id=23154077) J, [Werner-Wasik](https://pubmed.ncbi.nlm.nih.gov/?sort=date&term=Werner-Wasik+M&cauthor_id=23154077) M,  et al. [Lack of a dose-effect relationship for pulmonary function changes after stereotactic body radiation therapy for early-stage non-small cell lung cancer.](https://pubmed.ncbi.nlm.nih.gov/23154077/)  Int J Radiat Oncol Biol Phys. 2013 Mar 15;85(4):1074-81. doi: 10.1016/j.ijrobp.2012.09.016. Epub 2012 Nov 12. PMID: 23154077.
22. [Stone](https://pubmed.ncbi.nlm.nih.gov/?sort=date&term=Stone+B&cauthor_id=26334751) B,  [Mangona](https://pubmed.ncbi.nlm.nih.gov/?sort=date&term=Mangona+VS&cauthor_id=26334751) VS,  [Johnson](https://pubmed.ncbi.nlm.nih.gov/?sort=date&term=Johnson+MD&cauthor_id=26334751) MD, [Ye](https://pubmed.ncbi.nlm.nih.gov/?sort=date&term=Ye+H&cauthor_id=26334751) H, [Grills](https://pubmed.ncbi.nlm.nih.gov/?sort=date&term=Grills+IS&cauthor_id=26334751) IS . Changes in Pulmonary Function Following Image-Guided Stereotactic Lung Radiotherapy: Neither Lower Baseline nor Post-SBRT Pulmonary Function Are Associated with Worse Overall Survival. J Thorac Oncol.  2015 Dec;10(12):1762-9. doi: 10.1097/JTO.0000000000000670. PMID: 26334751.
23. **Stanic S, Paulus R, Timmerman RD, Michalski JM, Barriger RB, Bezjak A, et al.** [No clinically significant changes in pulmonary function following stereotactic body radiation therapy for early- stage peripheral non-small cell lung cancer: an analysis of RTOG 0236.](https://pubmed.ncbi.nlm.nih.gov/24661663/) Int J Radiat Oncol Biol Phys. 2014 Apr 1;88(5):1092-9. doi: 10.1016/j.ijrobp.2013.12.050. PMID: 24661663.

**Figure legends**

Figure 1. Cryoprobe 3.4 mm in diameter and a guide needle system consisting of a guide needle, inner sheath, and outer sheath.

Figure 2. Patient algorithm. SBRT, stereotactic body radiation therapy.

Figure 3. Local control curve. A 95% confidence interval is shown in shade.

Figure 4. Overall survival curve. A 95% confidence interval is shown in shade.

Figure 5. Recurrence-free survival curve. A 95% confidence interval is shown in shade.

Figure 6. Disease-specific survival curve. A 95% confidence interval is shown in shade.

Figure 7. Box plot comparison of forced expiratory in 1 s (FEV_1_) between before and 6 months after treatment, of which median values are 1.8 L (IQR, 1.5 － 2.1) and 1.7 L (IQR, 1.3 － 2.0); the latter is significantly lower than the former (p<0.001). The mean percentage of post-FEV_1_ /pre-FEV_1_ was 96 ±9% (median, 97; range, 77-119; IQR, 92-102).

Table 1. Patient characteristics

Characteristics	Value

Number of patients	66 (100%)

Age (y)	76 ± 7 (range: 58 – 89)

Sex	Male	42 (64)

Female	24 (36)

FEV_1_/FVC (%)	67 ± 13 (range: 33 - 89)

%FEV_1_	98 ± 31 (range: 38 - 184)

Charlson comorbidity index	2.1 ± 1.3 (range: 0 - 7)

Tumor histology

Adenocarcinoma	43 (65)

Squamous cell carcinoma	　　23 (35)

Tumor size (cm)	2.7 ± 0.7 (range: 2.0 – 5.4)

2 － 3	48 (73)

>3 － 4	15 (23)

>4 － 5	2 (3)

>5 － 6	1 (2)

T-stage

T1mi or T1a	　3 (5)

T1b	17 (18)

T1c	33 (48)

T2a	11 (17)

T2b	2 (3)

SUV on PET	5.0 ± 4.1 (range: 0.6 – 17)

FEV_1_/FVC: Forced expiratory volume in 1 second / forced vital capacity.

Table 2. Treatment details of stereotactic body radiation and cryoablation

SBRT

Number of fractions

Median (range)	5 (4–10)

IQR	5 – 5

Total dose (Gy)

Median (range)	50 (40–60)

IQR	44 –50

Biological effective dose (Gy)

Median (range)	100 (70–120)

IQR	82 –100

V20 (%)

Median (range)	3.1 (1.5–5.5)

IQR	2.4 – 4.0

Cryoablation

Number of cryoprobes used per procedure

Median (range)	1 (1–2)

IQR	1 – 1

Number of cycles per procedure

Median (range)	3 (3–5)

IQR	3–4

SBRT: stereotactic body radiation therapy; IQR: interquartile range

Table 3. Characteristics of patients who presented local recurrence

No. Age/Sex Tumor size Histology Local recurrence	Treatment	Prognosis (months)

1	70/F	3.1 cm	Sq	61 months	None	Dead with other disease (61)*

2	86/M	2.3 cm	Ad	33 months	None	Dead with disease (41)

3	68/M	2.8 cm	Sq	16 months	Surgery	Dead with disease (44)

4	58/M	2.6 cm	Ad	13 months	Chemotherapy Dead with disease (29)

5	78/M	3.0 cm	Sq	8 months	Surgery	Alive without disease (9)

M: male; F: female; Ad: adenocarcinoma; Sq: squamous cell carcinoma,

*: Local recurrence found at autopsy for other disease.

Table 4. Characteristics of patients who suffered pneumonia

Pneumonia

No. Age/Sex	Tumor size Type	Onset	Grade	Prognosis (months)

1	80/F	3.1 cm	BP	7 weeks	2	Dead with other disease (87)

2	79/F	2.2 cm	RP	9 weeks	3	Dead with other disease (61)

3	82/F	2.7 cm	RP	11 weeks	2	Dead with other disease (61)

4	80/M	4.2 cm	RP	12 weeks	2	Dead with disease (57)

5	82/F	5.0 cm	RP	13 weeks	2	Dead with disease (73)

6	77/F	2.4 cm	RP	15 weeks	2	Alive without disease (72)

Onset: onset time of pneumonia after stereotactic radiation therapy,

M: male; F: female; BP: broncho-pneumonia; RP: radiation pneumonitis.

Table 5. Cox proportional hazard regression analysis for local recurrence

Univariate analysis

Variables	HR (95% CI)	P

Tumor size	1.0 (0.3 － 3.5)	0.95

Tumor stage	1.5 (0.6 － 4.1)	0.39

Tumor histology (Ad vs. Sq)	3.2 (0.5 － 19.3)	0.20

SUV on PET	1.1 (0.9 － 1.3)	0.57

HR: hazard ratio; CI: confidence interval,

Ad: adenocarcinoma; Sq: squamous cell carcinoma,

SUV: standardized uptake value; PET: positron emission tomography.

Table 6. Cox proportional hazard regression analysis for overall survival

Univariate analysis	Multivariate analysis

Variables	HR (95% CI)	P	HR (95% CI)	P

Age	1.0 (1.0 － 1.1)	0.17	**―**

Sex	0.6 (0.2 － 1.7)	0.33	―

Comorbidity index	1.2 (0.9 － 1.5)	0.23	―

Tumor size	1.3 (0.8 － 2.0)	0.28	―

Tumor stage	1.7 (1.1 － 2.4)	0.01	1.5 (0.9 – 2.5)	0.09

SUV on PET	1.1 (1.0 － 1.2)	0.02	1.0 (1.0 – 1.1)	0.29

Tumor histology (Ad vs. Sq) 1.8 (0.9 － 3.8)	0.09	1.2 (0.5 – 2.7)	0.63

HR: hazard ratio; CI: confidence interval,

SUV: standard uptake value; PET: positron emission tomography,

Ad: adenocarcinoma; Sq: squamous cell carcinoma.

Table 7　Logistic regression analysis for posttreatment pneumonia

Univariate analysis	Multivariate analysis

Variables	OR (95% CI)	P	OR (95% CI)	P

Age	1.1 (1.0 － 1.3)	0.15	0.9 (0.8 - 1.0)	0.18

Comorbidity index	0.9 (0.5 － 1.7)	0.76	―

FEV_1_/FVC	1.0 (1.0 － 1.1)	0.59	―

Tumor size	2.1 (0.8 － 5.3)	0.11	0.6 (0.2 – 1.9)	0.36

V20	1.9 (0.8 － 4.6)	0.14	0.7 (0.3 － 2.0)	0.51

FEV1/FVC: Forced expiratory volume in 1 second / forced vital capacity.
