# Supplementary material for: Long-term outcomes of combination therapy with stereotactic body radiation therapy plus cryoablation using liquid nitrogen for stage I non-small cell lung cancer with tumors ≥2 cm
Source: PLoS One. 2025 Oct 8;20(10):e0332893. doi: 10.1371/journal.pone.0332893 (PMC12507226; doi:10.1371/journal.pone.0332893)
Supplement: Attachment — Submitted filename: 2025.6.7.Response to comments.docx [file pone.0332893.s007.docx]

June 6, 2025

[Editor’s name]　Dr. Xing-Xiong An, M.D

[Journal name」*PLOS ONE*

Dear Editor:

We wish to re-submit our revised manuscript titled, “Long-term outcomes of stereotactic body radiation therapy followed by cryoablation using liquid nitrogen for stage I non-small cell lung cancer with tumor size ≥2 cm.” The manuscript ID is PONE-D-25-05871.

We thank you and the reviewers for your thoughtful suggestions and insights. The manuscript has benefited from these helpful comments, and we look forward to working with you and the reviewers to move this manuscript closer to publication.

The manuscript has been rechecked and the necessary changes have been made in accordance with the reviewers’ suggestions. We hope these meet the approval of the reviewers. These corrections do not influence the content and framework of the paper. The responses to all comments have been prepared and attached given below.

We have marked them in **Red Bold type** in the marked version of manuscript.

We have also attached the non-marked one.

Thank you for your consideration. We look forward to hearing from you.

Sincerely,

Hiroaki Nomori, MD

Department of General Thoracic Surgery, Kashiwa Kousei General Hospital, 617 Shikoda, Kashiwa city, 277-8551, Chiba, Japan

Tel: +81-471-45-1111

E-mail: [hnomori@qk9.so-net.ne.jp](mailto:hnomori@qk9.so-net.ne.jp)

Response to reviewer

The corrected points were in marked paper with Red Bold type.

1. Novelty and Contextualization

The rationale for combining SBRT and cryoablation is interesting but not fully contextualized in relation to prior combination therapy reports (if any exist). A more explicit explanation of how this study is novel compared to previous SBRT or ablation-alone studies is warranted.

➡　From 37 – 49: We rewrote the Objectives in the abstract as follows: **While radiation and ablation therapy have been performed for stage I non-small cell lung cancer, their local control and survival rates are not satisfactory for tumors ≥2 cm. This study administered a combination therapy of stereotactic body radiation and cryoablation and evaluated its effectiveness based on 5-year survival rates in patients with stage I non-small cell lung cancer ≥2 cm.**

While the radiation or ablation therapy has been conducted for T1N0M0 lung cancer, most of their survival have been examined with 3-year, which is not long enough. In fact, the 5-year survival of stereotactic body radiation for stage I lung cancer was reportedly decreased to 40%. This study aimed to assess 5-year treatment results with combination therapy of stereotactic body radiation and cryoablation for T1-2N0M0 non-small cell lung cancer with tumor size ≥2 cm with 73 months of median follow-up.

➡From line 121 – 131: We wrote the following:

**However, the LC rates after both cryoablation and RFA are reportedly lower for NSCLCs with tumor size ≥2 cm than those <2 cm [10,11], consistent with SBRT results [9].**

**The mechanism of tumor control differs for SBRT, cryoablation, and RFA. SBRT causes injury to the tumor DNA. Cryoablation induces the formation of ice crystals, leading to cell membrane destruction and subsequent intracellular dehydration. RFA leads to coagulation necrosis owing to high temperatures. Due to the different mechanisms, a combination of these therapies may facilitate improve LC for stage I NSCLC with tumor size ≥2 cm; however, this combination therapy has not been explored.**

2. Patient Selection

- The study mixes patients who were medically inoperable and those who preferred non-surgical treatment. This creates heterogeneity in the cohort. Please discuss how this choice affects generalizability and whether outcomes differ between these two groups.

➡From line 368 – 370: We wrote the following:

**Although the study included both operable and inoperable patients, the OS exhibited no correlations with age and comorbidity, indicating the generalizability of the SBRT-cryoablation treatment for both patient groups.**

- Inclusion of two patients with stage II NSCLC (T2bN0M0) should be acknowledged and justified more explicitly or excluded for consistency with the stated focus on Stage I disease.

➡ We deleted 2 patients with T2b, i.e., stage II, which changed the manuscript as follows:

From line 1 – 3: Title was changed to the following:

“**Long-term outcomes of stereotactic body radiation therapy followed by cryoablation using liquid nitrogen for stage I non-small cell lung cancer with tumor size ≥2 cm”**.

From line 37 – 41: The abstract was changed to the following:

**While radiation and ablation therapy have been performed for stage I non-small cell lung cancer, their local control and survival rates are not satisfactory for tumors ≥2 cm. This study administered a combination therapy of stereotactic body radiation and cryoablation and evaluated its effectiveness based on 5-year survival rates in patients with stage I non-small cell lung cancer ≥2 cm.**

While the radiation or ablation therapy has been conducted for T1N0M0 lung cancer, most of their survival have been examined with 3-year, which is not long enough. In fact, the 5-year survival of stereotactic body radiation for stage I lung cancer was reportedly decreased to 40%. This study aimed to assess 5-year treatment results with combination therapy of stereotactic body radiation and cryoablation for T1-2N0M0 non-small cell lung cancer with tumor size ≥2 cm with 73 months of median follow-up.

From line 50-52: The number of patients was changed as “**64 patients with stage I non-small cell lung cancer ≥2 cm 66 patients with T1-2N0M0 non-small cell lung cancer ≥2 cm”**

Line 58: The median follow-up duration was changed from 73 to **74** months.

Line 59; The mean tumor diameter was change to **2.7 ± 0.5 0.7 (range: 2.0–4.0 5.4) cm.**

Line 61: The 5-year overall survival was changed from 75 to **74%**.

Line 63: post-cryoablation pneumothorax was changed from 38 to **40%**.

Line 64: The number of patients with pneumonitis was changed from 6 (9%) to **“Five (8%)”**

Line 65: The number of patients with radiation pneumonitis was changed from 5 to **4**.

Line 66: The number of patients with grade 2 pneumonitis was changed from five to **four**.

Line 69: T1-2N0M0 was change to **stage I**.

Line 145 - 146: The number of patients was changed to **64 patients with stage I 66 patients with cT1-2N0M0** .

Line 148: Median follow-up period was changed from 73 to **74** months.

Line from 278 – 286: The patient algorithm was changed as the following:

**Between 2015 and 2023,** **220 patients with stage I NSCLC with tumor size ≥2 cm underwent local treatments, including surgery, SBRT, and cryoablation (Fig. 3). Among the 78 patients who underwent cryoablation, 64 patients were treated with** **the SBRT-cryoablation regimen** (**Table 1**). **333 patients with cT1-2N0M0 non-small cell lung cancer with tumor size ≥2 cm underwent local treatments, including surgery, SBRT, and cryoablation (Fig. 2). Among 80 patients who underwent cryoablation, 66 patients, including 2 patients with T2bN0M0 (Stage II), were treated by SBRT followed by cryoablation (Table 1).**

Line from 291 – 292: It was changed as the following: “The FDG-PET was conducted for **61 of the 64 patients (95%) 62 of the 66 patients (94%).**”

Line from 298 – 299: It was changed as the following: **The median follow-up period was** **74 (range, 3–111; IQR, 41–89) months**. **73 months (range, 3–111; IQR, 42–88).**

Line from 303 – 305: It was changed as the following: “**The 3- and 5-year OS rates were 87% (CI,** **78–95% 79%–96%) and** **74% (CI,** **63–86% 64%–86%), respectively (Fig. 5 4). During the follow-up period, 31 (48%) 32 (48%)died, of which 23 24 died due to other diseases.**

Line from 306 – 309: The RFS was changed as the following: “**The 3- and 5-year RFS rates were 74% (CI,** **63–85% 63%–84%) and 60% (CI, 48–73%), respectively (Fig. 6 5).** **Twelve patients (14%) Fifteen patients (23%) experienced distant metastases between 3 and 60 months after treatment.**

Line from 310 – 311: The DSS was changed as “The 3- and 5-year DSS rates were **96% (CI, 92–100%) and 92% (CI, 85–100%) 97% (CI, 92%–100%) and 93% (CI, 86%–100%), respectively (Fig. 7 6).**“

Line from 313 – 319: It was changed as the following**: Pneumothorax** **with CTCAE grade 2 occurred in 26 (40%) in 25 (38%) patients patients after cryoablation, with a median chest tube drainage period of 2 days (IQR, 1–4). Median duration of hospital stay after cryoablation was 5 days (IQR, 4–5). Five (8%) Six (9%) patients developed pneumonia with CTCAE grade 2 (n=4) and grade 3 (n=1) between 7 and 15 weeks after treatment (Table 4). Among them, four five had radiation pneumonitis and one had bronchopneumonia. ”**

Line from 334 – 339: It was changed as the following: “Pulmonary function tests were performed before and 6 months after treatment in **59 of 64 patients (92%) 61 (92%) 66 patients**. A significant decrease in FEV_1_ was observed between pretreatment and posttreatment (1.8 ± 0.6 vs. 1.7 ±**0.5 0.6** L, P < 0.001), of which the mean post-/pre-FEV_1_ percentage was **95 ± 10% (median, 96; IQR, 90–100) (Fig. 8). 96 ± 9% (median, 97; IQR, 92–102) (Fig. 7)**.

From line 342 – 346: It was changed as “**This study highlights the following points: (1)** **the 5-year OS after SBRT-cryoablation regimen for stage I NSCLC ≥2 cm was better than that reported in previous studies on SBRT alone for stage I NSCLC including tumors < 2 cm 5-year OS after SBRT followed by cryoablation was better than that reported in studies on SBRT alone**; ”

Line 348-350: It was changed as “ (4) **pneumothorax after cryoablation was observed in 40% 38% of the patients, of which median drainage period was 2 days.**

3. SBRT Technique Clarification

➡From 175 – 207: SBRT technique was written in detail as follows: “**SBRT was administered using an Elekta Versa HD (Elekta Co. Ltd., Tokyo, Japan).** **Respiratory motion was managed using three sets of conventional free-breathing CT simulation images, with an abdominal compression device for breath-hold.**

**Internal tumor volume (ITV), encompassing the full range of tumor motion, was delineated using the second and the third image sets and referenced to the clinical target volume (CTV) defined in the first image set. A planning target volume (PTV) margin of 5 mm was added to the ITV based on the Radiation Therapy Oncology Group (RTOG) studies 0236, 0813, and 0915.**

**Fractionation schemes were determined by radiation oncologists to optimize the PTV coverage while adhering to organ-at-risk dose constraints based on the RTOG protocols. Each fractionation schedule delivered a biological effective dose (BED) of at least 80 Gy (α/β=10 Gy).**

**Treatments were administered once daily on consecutive days. Before each irradiation session, a flat-panel cone-beam CT scan was performed to identify the tumor location. This was compared to the CTV from the treatment plan. If a positional discrepancy was detected, corrections were made using a six-degree-of-freedom treatment couch or manually.**

**Respiratory motion was managed using three sets of conventional free-breathing CT simulation images, with or without the use of a breath limitation device.**

**Internal tumor volume encompassing the full range of tumor motion was delineated using the second and the third images of the clinical target volume of the first image. A planning target volume expansion of 5 mm was applied to the internal tumor volume. Fractionation schemas were determined by radiation oncologists to optimize the planning target volume coverage and meet organ-risk dose constraints based on Radiation Therapy Oncology Group (RTOG) 0236, 0813, and 0915 trials. Each fractionation schedule contained a biological effective dose (BED) of at least 80 Gy (α/β=10). Treatment courses were administered using a consecutive-day, one-daily fractionation schedule.**

- The SBRT section lacks clarity on how tumor motion was handled quantitatively. Was 4D-CT used? Are the three free-breathing scans equivalent to a poor-man's ITV?

➡All CT scans used in this study were volumetric (3D) scans, which capture the tumor three-dimensionally. While we did not use 4D-CT, we believe that acquiring and fusing three separate CT scans taken at different times can serve as a practical substitute for assessing tumor motion over time. To minimize respiratory variability and avoid a so-called “poor-man’s ITV,” patients were instructed to maintain consistent breathing during all scans. This instruction is a standard procedure at all treatment facilities involved and was therefore not specifically mentioned in the manuscript.

- The use of a 5 mm margin to define PTV may be standard, but justification or comparison with motion management strategies (e.g., gating, abdominal compression) would be helpful.

➡We used abdominal compression with a belt to reduce respiratory motion.

4. Statistical Analysis

- Multivariate analyses were conducted despite small event numbers (e.g., only 6 pneumonia events). Please clarify how overfitting or type II errors were mitigated, or consider simplifying the analysis or using Firth’s correction where applicable.

➡　From 330 – 333: We deleted the analysis for pneumonia and Table 7 as follows: **Univariate logistic regression analysis for pneumonia showed correlations (p≤0.15) with age (p=0.15), tumor size (p=0.11), and V20 (p=0.14); however, these correlations were not significant in the multivariate analysis (p=0.18–0.51) (Table 7).**

- The use of Microsoft Excel 10 for statistical analysis may not inspire confidence in reproducibility. Consider using a more robust statistical platform (e.g., R, SPSS, or Stata) or at least justify the use of Excel.

➡ From line 273 – 275: We used SPSS for statistical analysis as “**Statistical analyses were performed using** **SPSS Statistics version 29 (IBM, NY, USA) using Microsoft Excel version 10 (Redmond, WA, USA).”**

5. Reported events

- There is no grading of pneumothorax severity. Consider including this to align with CTCAE standards.

➡From line 313 – 315: We added the pneumothorax severity as “**Pneumothorax with CTCAE grade 2 occurred in 26 (40%) in 25 (38%) patients** patients after cryoablation, with a median chest tube drainage period of 2 days (IQR, 1–4).”

- The decrease in FEV1 is statistically significant but arguably not clinically meaningful. This distinction should be emphasized.

➡From line 382 – 383: We wrote the reason for examining decrease in FEV1 as “**To clarify severity of lung damage, the pulmonary function was assessed both before and after treatment.**”

While the manuscript aims to compare cryoablation and SBRT for early-stage NSCLC, it currently lacks sufficient detail regarding the cryoablation technique. For the study to be scientifically rigorous and clinically informative, it is important that the authors provide a comprehensive description of the cryoablation procedure. This should include, but not be limited to probe placement strategy, imaging modality for guidance, lesion size eligibility, and criteria for defining adequate ablation margins. Without this information, readers are unable to fully assess the comparability of the two treatment modalities, and the reproducibility of the cryoablation approach is compromised. I recommend that the authors include this information in the revised manuscript.

➡　Please see our reply for this in the next part.

6. Lack of details on the Cryoablation Technique

There is no information on imaging guidance used, lesion size limitation, or margin assessment. Without understanding how cryoablation was applied, it’s difficult for readers to assess if the outcomes were influenced by technical variability, operator experience, or treatment adequacy.

➡From line 217 – 225: We wrote the detailed technique of cryoablation as “**The patient was placed in the supine or prone position on the CT table, depending on the tumor location. Sedatives such as pethidine hydrochloride (35 mg) and midazolam (2–3 mg) were administered. Following local anesthesia, the guide needle was introduced into a tumor under CT guidance (Fig. 2). The inner and outer sheaths were advanced over the guide needle to reach the tumor. Once the outer sheath reached the target position, the guide needle and inner sheath were removed. The cryoprobe was then introduced into the outer sheath, and freezing commenced.”**

➡　We also added the CT views for placement the cryoablation and the CT 5 months after the treatment ) in Fig.2.

_________________________________________________________________________

Minor Comments

1. Language and Style

- The text is mostly clear but would benefit from professional editing for grammar, flow, and tense consistency (e.g., “conducted the cryoablation” → “cryoablation was performed”).
- “Study treatment” is vague and repetitive. Consider replacing with “combination therapy” or “SBRT-cryoablation regimen” for clarity.

➡While the previously submitted manuscript was already revised by native speakers, we have made one more English revision. Combination therapy was changed to “**SBRT-cryoablation treatment**” throughout the manuscript.

2. Formatting

- Ensure consistent use of abbreviations (e.g., CTCAE, SUV, BED) and define them on first use.

➡ Line 240-241: We wrote “**Common Terminology Criteria for Adverse Events (CTCAE version 4.03).”**

➡Line 252-253: We wrote “**Accumulation on FDG-PET was evaluated using** **a maximum** **standardized uptake value (SUV).**”

➡Line 188: We wrote” **biological effective dose (BED).**”

- The phrase “on the other hand” is overused in the Introduction. Rephrasing or restructuring is recommended for better logical flow.

➡ Line 117: We deleted “On the other hand”.
