# Supplementary material for: Long-term outcomes of combination therapy with stereotactic body radiation therapy plus cryoablation using liquid nitrogen for stage I non-small cell lung cancer with tumors ≥2 cm
Source: PLoS One. 2025 Oct 8;20(10):e0332893. doi: 10.1371/journal.pone.0332893 (PMC12507226; doi:10.1371/journal.pone.0332893)
Supplement: Attachment — Submitted filename: Re-revised_comments.docx [file pone.0332893.s008.docx]

Thank you for the opportunity to review this revised manuscript entitled “Long-term outcomes of stereotactic body radiation therapy followed by cryoablation using liquid nitrogen for stage I non-small cell lung cancer with tumor size ≥2 cm.”

I commend the authors for their thorough revision in addressing the reviewers’ previous comments and for the scientific merit of this study, which explores an important combination therapy approach for early-stage NSCLC. The manuscript demonstrates solid scientific content, with clear objectives and appropriate methodology.

However, I note that the manuscript does not yet fully meet journal writing standards in terms of its scientific writing presentation. Specifically:

- Tables and figures are cited within the text but lack adequate integration and explanation. Standard journal writing requires that each table and figure is not only referenced but also introduced with context, summarised with key findings, and interpreted within the Results and Discussion. Currently, many citations simply direct the reader to the figure or table without providing an explanation of its relevance or implications.
- Despite native revision, the language and grammar remain awkward in several sections, with phrasing that may impede clarity for an international readership. Further professional language editing is required to ensure fluency, precision, and consistency.
- The results presentation tends to read as raw data reporting rather than a scientific narrative, which is expected in journal publications to guide readers through the study findings and their implications systematically.

I recommend major language editing and revision of the Results and Discussion sections, with attention to:

- Introducing each table and figure with purpose.
- Explaining what each demonstrates.
- Interpreting how each finding addresses the study objectives and contributes to the field.

Overall, I believe that with these revisions, the manuscript will reach the presentation standards required for publication and effectively communicate its important findings to the wider oncology and radiology community.

| **General comments**  Based on the manuscript reviewed, while it demonstrates solid scientific content, it does not fully meet journal writing standards in terms of scientific writing presentation   - The manuscript often lists results without interpretation or context. - Tables and figures are cited but not integrated or explained clearly within the narrative. - For example, “Table 2 presents the treatment details” simply points to the table without summarising key findings. - Example: “Both the 3- and 5-year LC rates were 93% (Fig. 4).” - Language, although revised, retains awkward phrases and grammar issues.   Examples:   - “Due to the different mechanisms, a combination of these therapies may facilitate improve LC…” - “which would decrease OS after SBRT.” - In the provided text, captions or explanations for figures were not included below each figure. The manuscript references figures (e.g. Fig. 1, Fig. 2, Fig. 3) within the text body, describing their content, but the actual figure legends or captions themselves were not shown in the extracted PDF text. | |
| --- | --- |
| **Pg, Line** | **Specific Comments** |
| P9, line 156- 157 | …the use of a breath limitation device.   - Could you please clarify – are you referring to the abdominal compression device mentioned earlier, or is this referring to a different breath-hold or respiratory management device? |
| P10,line 161 | SBRT was administered using an Elekta Versa HD (Elekta Co. Ltd., Tokyo, 11 Japan)…   - For clarity, please specify that the Elekta Versa HD is a linear accelerator. |
| P11, line: 166 – 167 | … referenced to the 167 clinical target volume (CTV) defined in the first image set   - Could you please clarify – is this referencing the CTV or the GTV? Typically, the ITV is delineated with reference to the gross tumor volume (GTV) rather than the CTV. |
| P11, line 176 | …identify the tumor location   - For clarity, please note that cone-beam CT (CBCT) is used to verify the isocentre and tumor positioning, ensuring that the GTV is encompassed within the ITV as planned. Consider revising this in the methods description. |
| **Overall assessment:**   - Scientifically valid but lacking in polished presentation. - Reads more like a technical report than a high-quality journal article. | |
| **Recommendation:**   - Major language editing and rewriting of Results and Discussion to: - Integrate explanations for figures and tables. - Enhance clarity and scientific storytelling. - Refine language and grammar for journal-level fluency. | |
