# Supplementary material for: Long-term outcomes of combination therapy with stereotactic body radiation therapy plus cryoablation using liquid nitrogen for stage I non-small cell lung cancer with tumors ≥2 cm
Source: PLoS One. 2025 Oct 8;20(10):e0332893. doi: 10.1371/journal.pone.0332893 (PMC12507226; doi:10.1371/journal.pone.0332893)
Supplement: Attachment — Submitted filename: renamed_88f25.docx [file pone.0332893.s009.docx]

Ms. Ref. No.: PONE-D-25-05871

Dear Editor in chief,

Long-term outcomes of combination therapy with stereotactic body radiation therapy and cryoablation for T1-2N0M0 non-small cell lung cancer with tumor size ≥2 cm

Dear Editor,

I appreciate your valuable comments for our paper.

I have revised the paper, i.e., **marked one**, in which corrected points are written in **Bold style**.

The responses to the comments for the reviewer with point-by-point are as follows:

- **Response to general comment:**

**Comment 1**: The manuscript often lists results without interpretation or context.

**Response 1**: Tables and figures are explained in each section.

**Line 323 – 330:** Re: Table 1. **--- (Table 1).** **The reasons for selecting the SBRT-cryoablation treatment included a Charlson comorbidity index ≥3 or FEV_1_/FVC <70% or age ≥80 years in 28 patients (44%); 36 (56%) patients requested the SBRT-cryoablation treatment rather than surgery without those unfavorable factors. Tumor size shows the maximum size including ground glass opacity area, of which tumor stage was T1mi or T1a in 3 patients, T1b in 17, T1c in 32, and T2a in 12. FDG-PET was performed in 61 of the 64 patients (95%).**

**Line 340 – 344:** Re: Table 2. **Table 2** shows the treatment details. SBRT was administered with median fractions of 5 (interquartile range (IQR), 5–5), a median total dose of 50 Gy (IQR, 44–50), and a median BED of 100 Gy (IQR, 82–100). Cryoablation was **performed** conducted with a median number of 1 probe (IQR, 1–1) and 3 cycles (IQR, 3–4).

**Line 352 – 384**: Re: Figure 4 a-d. **The 3- and 5-year LC rates were both 93% (95% confidence interval [CI], 87–100%) (Fig. 4a). The median LC was 73 (range, 3–110; IQR, 36–87) months. Local recurrence was diagnosed in 5 (8%) patients, with a median LC of 16 (range, 8–61; IQR, 13–34) months (Supplement Table 1).**

Both the 3- and 5-year LC rates were 93% (95% confidence interval [CI], 87%–100%) (**Fig. 3**). Local recurrence was diagnosed in 5 (8%) patients between 8 and 61 months after the treatment (**Table 3**).

**The 3- and 5-year OS rates were 87% (CI, 78–95%) and 74% (CI, 63–86%), respectively (Fig. 4b). The median OS was 74 (range, 3–110; IQR, 41–89) months. During the follow-up period, 31 (48%) patients died, of which 7 died of the primary disease and 24 died of other diseases. Of the latter 24 patients, 18 patients died of non-cancer diseases, 5 died of cancer of other organs, and 1 died of secondary lung cancer. Although the median OS of the 7 patients who died of the primary disease was 44 (range, 24–89; IQR, 35–65) months, that of the 24 patients who died of other diseases was 60 (range, 3–96; IQR, 33–73) months.**

The 3- and 5-year OS rates were 87% (CI, 79%–96%) and 75% (CI, 64%–86%), respectively (**Fig. 4)**. During the follow-up period, 32 (48%) patients were dead, with 24 of them dying of other diseases.

**The 3- and 5-year RFS rates were 74% (CI, 63–85%) and 60% (CI, 48–73%), respectively (Fig. 4c). The median RFS was 64 (range, 3–110; IQR, 32–82) months. The median RFS of the 15 relapsed patients was 29 (range, 3–96; IQR, 33–73) months, whereas that of the 49 non-relapsed patients was 75 (range, 3–110; IQR, 44–89) months.**

The 3- and 5-year RFS rates were 74% (CI, 63%–84%) and 60% (CI, 48%–73%), respectively (**Fig. 5**). Fifteen patients (23%) experienced distant metastases between 3 and 60 months after the treatment.

**The 3- and 5-year DSS rates were 96% (CI, 92–100%) and 92% (CI, 85–100%), respectively (Fig. 4d). The median DSS was 74 (range, 3–110; IQR, 41–89) months. The median DSS of the 7 patients who died of the primary disease was 53 (range, 24–89; IQR, 38–64) months, whereas that of the 57 patients who died of other diseases was 77 (range, 3–110; IQR, 44–89) months.**

**Line 408 - 417**: **The** Univariate cox regression analysis **for the correlation of OS revealed significance with tumor stage, SUV on PET, and BED (P = 0.02, each). In the multivariate analysis, tumor stage and BED were significant (P = 0.04 and 0.02, respectively) (Table 3). Although the ROC curve analysis on the tumor stage and BED revealed that the optimal cut-off values were T1c and 81 Gy, respectively, the area under curve for both was < 0.7 (0.67 and 0.58, respectively).**

**Comment 2**: Tables and figures are cited but not integrated or explained clearly within the narrative

**Response 2:** Same as the Response 1.

**Comment 3:** Language, although revised, retains awkward phrases and grammar issues. The results presentation tends to read as raw data reporting rather than a scientific narrative, which is expected in journal publications to guide readers through the study findings and their implications systematically.

**Response 3**: The paper was revised by native speakers once again, of which certification was attached as supplement one.

**Comment 4:** In the provided text, captions or explanations for figures were not included below each figure. The manuscript references figures (e.g. Fig. 1, Fig. 2, Fig. 3) within the text body, describing their content, but the actual figure legends or captions themselves were not shown in the extracted PDF text.

**Response 4**: The legends of each figure was written as follows:

**Line 646 – 677**:

Figure 1. Cryoprobe**,** 3.4 mm in diameter**,** and a guide needle system consisting of a guide needle, inner sheath, and outer sheath.

**Figure 2. Cryoablation procedure. (a) Tumor after stereotactic body radiation therapy in the dorsal segment of the left upper lobe (indicated by an arrow); (b) The outer sheath penetrated the tumor; (c) The cryoprobe was introduced into the outer sheath, followed by cryoablation; (d) Five months after the treatment.**

Figure **3** 2. Patient algorithm. **Of the 220 patients with non-small cell lung cancer with stage I ≥2 cm, 78 patients were treated by cryoablation, of which 64 were treated by SBRT followed by cryoablation.** SBRT, stereotactic body radiation therapy.

**Figure 4. Kaplan-Meier curves. The 95% confidence interval is depicted in the shaded area. (a) Local control. The 3- and 5-year local control rates were both 93%.; (b) Overall survival. The 3- and 5-year overall survival rates were 87% and 74%, respectively.; (c) Recurrence-free survival. The 3- and 5-year recurrence-free survival rates were 74% and 60%, respectively.; (d) Disease-specific survival. The 3- and 5-year disease-specific survival rates were 96% and 92%, respectively.**

Figure 3. Local control curve. A 95% confidence interval is shown in shade.

Figure 4. Overall survival curve. A 95% confidence interval is shown in shade.

Figure 5. Recurrence-free survival curve. A 95% confidence interval is shown in shade.

Figure 6. Disease-specific survival curve. A 95% confidence interval is shown in shade.

**Supplement Figure 1.** Figure 7. Box plot comparison of forced expiratory in 1 s (FEV_1_) between before and 6 months after treatment. **A significant decrease in FEV_1_ was observed between pretreatment and posttreatment (1.8 ± 0.6 vs. 1.7 ± 0.5 L, P < 0.001), with a mean post-/pre-FEV_1_ percentage of 95 ± 10% (median, 96; IQR, 90–100)**. , of which median values are 1.8 L (IQR, 1.5 － 2.1) and 1.7 L (IQR, 1.3 － 2.0); the latter is significantly lower than the former (p<0.001). The mean percentage of post-FEV_1_ /pre-FEV_1_ was 96 ±9% (median, 97; range, 77-119; IQR, 92-102).

**Comment 5**: P9, line 156- 157. …the use of a breath limitation device.

**Response 5:** The breath limitation device was explained as follows:

**Line 207 – 210:** Respiratory motion was managed using three sets of conventional free-breathing CT simulation images, **with an abdominal compression device for breath-hold (Pressure Belt; Euro Meditech Co., Ltd., Tokyo, Japan).** with or without the use of a breath limitation device.

**Comment 6:** P10,line 161. SBRT was administered using an Elekta Versa HD (Elekta Co. Ltd., Tokyo, 11 Japan). For clarity, please specify that the Elekta Versa HD is a linear accelerator.

**Response 6:** We rewrote it as follows:

**Line 206 – 207:** SBRT was administered **using a linear accelerator,** Electa Versa HD (Electa Co. Ltd., Tokyo, Japan).

**Comment 7:** P11, line: 166 – 167. referenced to the 167 clinical target volume (CTV) defined in the first image set. Could you please clarify – is this referencing the CTV or the GTV? Typically, the ITV is delineated with reference to the gross tumor volume (GTV) rather than the CTV.

**Response 7:**

We used the term of the gross tumor volume (GTV) instead of the clinical target volume (CTV), because, the CTV is the same volume as the GTV in this study. It is not explained in the text.

**Comment 8:** P11, line 176. identify the tumor location. For clarity, please note that cone-beam CT (CBCT) is used to verify the isocentre and tumor positioning, ensuring that the GTV is encompassed within the ITV as planned. Consider revising this in the methods description.

**Response 8:** We changed the sentences as follows:

**Line 211 – 230**: Internal tumor volume **(ITV),** encompassing the full range of tumor motion, was delineated using the second and the third **image sets** images of and **referenced** to the clinical target volume **(CTV) in** of the first image **set**. **A planning target volume (PTV) margin of 5 mm was added to the ITV based on the Radiation Therapy Oncology Group (RTOG) studies 0236,0618, and 0915 [5,6,8].** expansion of 5 mm was applied to the internal tumor volume.

Fractionation schemas were determined by radiation oncologists to optimize the **PTV** planning target volume coverage **while adhering to** and meet organ**-at-**risk dose constraints based on the RTOG protocols. **Although each fractionation schedule usually delivered a biological effective dose (BED) of 100 Gy (α/β=10 Gy), it was lowered to a minimum of 80 Gy in patients ≥80 years old or who had interstitial pneumonitis on CT image.** Each fractionation schedule contained a biological effective dose (BED) of at least 80 Gy (α/β=10).

Treatments courses were administered **once daily** **on** using a **consecutive days,** one-daily fractionation schedule. **Before each irradiation session, a flat-panel cone-beam CT scan was performed to identify the tumor location. This was compared to the CTV from the treatment plan. If a positional discrepancy was detected, corrections were made using a six-degree-of-freedom treatment couch or manually.**

**Response to the comment of reviewer #1:**

**Comment 1:** Please, clarify more precisely how internal margin was defined (lines 158-160) and explain the rationale for accepting a BED <100Gy.

**Response 1:** I explained it as follows:

**Line 213 – 216: A planning target volume (PTV) margin of 5 mm was added to the ITV based on the Radiation Therapy Oncology Group (RTOG) studies 0236,0618, and 0915 [5,6,8].** expansion of 5 mm was applied to the internal tumor volume.

**Line 219 – 224: Although each fractionation schedule usually delivered a biological effective dose (BED) of 100 Gy (α/β=10 Gy), it was lowered to a minimum of 80 Gy in patients ≥80 years old or who had interstitial pneumonitis on CT image.** Each fractionation schedule contained a biological effective dose (BED) of at least 80 Gy (α/β=10).

**Comment 2:** Since bias related to observational and retrospective nature of the study is highlighted, it is bold to include a comparation with results of a prospective study on SBRT in the conclusion.

**Response 2:** In the conclusion, we rewrote as follows:

**Line 512 – 519:** **The SBRT-cryoablation treatment is a feasible treatment for stage I NSCLC ≥2 cm. Because the study was a single-arm cohort study, it cannot be compared with the studies of SBRT alone, all of which were also single-arm ones. This study may contribute to increasing treatment options in patients with stage I NSCLC ≥2 cm.** SBRT followed by cryoablation is a feasible treatment option for T1-2N0M0 non-small cell lung cancer with tumor size ≥2 cm, with improved survival and acceptable rates of adverse events, compared to stereotactic body radiation alone.

**Comment 3:** Moreover, I suggest emphazing the small size of cohort in the discussion.

**Response 3:** We rewrote it as follows:

**Line 505 – 506:** This study has the following limitations: (1) the retrospective descriptive design **with only 64 patients** and---.

**Response to the comment of reviewer #2:**

**Comment 1:** There is one patient with a T3 tumour (>5cm). If the inclusion is T1-T2, I am unsure why this patient was included. I suggest excluding T3 tumours.

**Response 1:**

I confined the study case as Stage I NSCLC with tumor size ≥2 cm, i.e., excluding 2 patients with T2b, as showing in Table 1. As the result, the number of cases were change from 66 to 64 patients.

**Comment 2:** A BED of at least 80 Gy will be considered low according to the current recommendation of at least 100 Gy, as outcomes tend to be poorer at doses less than 100 Gy. It would be beneficial to discuss the reasons why some tumours were treated with doses below 100 Gy. It is unclear whether the dose was reduced to facilitate combined treatment or to meet OAR dose constraints.

**Response 2:** We wrote the patients who were treated with <100 Gy of BED as follows:

**Line 219 – 224: Although each fractionation schedule usually delivered a biological effective dose (BED) of 100 Gy (α/β=10 Gy), it was lowered to a minimum of 80 Gy in patients ≥80 years old or who had interstitial pneumonitis on CT image.** Each fractionation schedule contained a biological effective dose (BED) of at least 80 Gy (α/β=10).

**Comment 3:** The follow-up CT scans ceased at three years (page 11, line 181), despite a median follow-up of 73 months. No information has been provided regarding the methods used to diagnose recurrences after this period.

**Response 3:** The follow-up was not ceased at three years. I wrote it more clearly as follows:

**Line 260 – 264:** Follow-up using CT was conducted every 3 or 4 months until 3 years after treatment, **with a minimum 6-month interval subsequently applied until the final follow-up day.** with a minimum 6-month interval being subsequently applied. In a case of suspected local recurrence on CT images, needle aspiration biopsy was performed.

**Comment 4:** Local recurrences were diagnosed either through needle biopsy or when “the primary site showed apparent increase” (page 12, line 208-9). Different patterns of fibrosis, including dense mass-like consolidation, can be observed after SBRT, making the criteria used in the study for radiologically diagnosing recurrence inadequate. It is necessary to provide a clear description of the criteria used for radiological diagnosis of recurrence when a needle biopsy was not performed, including the criteria used to select patients for needle biopsy and how radiation pneumonitis was differentiated from recurrence

**Response 4:** Yes, you are right, that is the reason why the LC (local control) after SBRT is unreliable. I wrote as follows:

**Line 447 – 451: The hidden tumor in the scar could cause distant metastases. Therefore, the high LC rates after SBRT, including that in the present study, may be unreliable. Treatment outcomes after SBRT should be evaluated based on OS, not on LC.** Therefore, treatment outcomes after SBRT should be evaluated based on survival rates.

**Comment 5:** In the regression analysis of factors correlating with LC and survival, BED of SBRT should be included, since it has been shown to affect these outcomes.

**Response 5:**

Thank you for your valuable comment. I added the BED for the analysis of LC and OS, which showed the significance of BED for OS. We rewrote the paper as follows:

**Line 302 – 307**: Univariate cox proportional hazards regression analysis **for the correlation of LC was used to assess tumor size, tumor stage, tumor histology, SUV on PET, and BED.** was used to assess variables related to LC and survival. **Univariate logistic regression analysis for the correlation of OS was used to assess age, sex, comorbidity index, tumor size, tumor stage, SUV on PET, tumor histology, and BED.**

**Line 402 – 417**:

**The** Univariate cox regression analysis **for the correlation of LC revealed that tumor size, tumor stage, tumor histology, SUV on PET, and BED were not significant (P = 0.22 – 0.63) (Supplement Table 3).** for LC revealed no correlations with tumor size, tumor stage, tumor histology (adenocarcinoma vs. squamous cell carcinoma), and SUV on PET (p=0.20 – 0.95) (**Table 5**).

**The** Univariate cox regression analysis **for the correlation of OS revealed significance with tumor stage, SUV on PET, and BED (P = 0.02, each). In the multivariate analysis, tumor stage and BED were significant (P = 0.04 and 0.02, respectively) (Table 3). Although the ROC curve analysis on the tumor stage and BED revealed that the optimal cut-off values were T1c and 81 Gy, respectively, the area under curve for both was < 0.7 (0.67 and 0.58, respectively).** for OS revealed correlations (p≤0.15) with tumor stage (p=0.01), SUV on PET (p=0.02), and tumor histology (p=0.09); however, these correlations were not significant in the multivariate analysis (p=0.09–0.63) **(Table 6)**.

**Line 469 – 474:** **The multivariate Cox regression analysis demonstrated the significance of BED with OS, with a cutoff value of 81 Gy. The patients treated with BED lower than 100 Gy were older than 80 years or had interstitial pneumonitis on CT. Although the area under curve on ROC was 0.58, which indicated low accuracy, the desirable BED would be close to 100 Gy.**

**Comment 6:** It has been noted that the majority of SBRT studies do not report results beyond three years, which is accurate. However, RTOG 0915 and RTOG 0236 have presented 5-year outcomes, with LC rate of 89.4-93.2%, and RTOG 0618 presented a 4-year LC of 96%. The local control rates observed in these RTOG studies are comparable to those reported in this study. I suggest discussing the results within the context of the outcomes of the RTOG studies.

**Response 6:**

**Line 124 – 129:** I added the followings: **The RTOG 0618 trial reported a 4-year OS rate of 56% for SBRT in 26 patients with T1–T2 N0M0 NSCLC [5]. The RTOG 0236 study reported a 5-year OS rate of 40% in 55 patients with inoperable stage I NSCLC [6].** The study conducted by Timmerman et al. on SBRT for stage I NSCLC showed 56% of 3-year study [2], which was decreased to 40% in their 5-year assessment [6].

**Line 456 – 460**: I added the followings: **The RTOG 0915 and RTOG 0236 studies and Shibamoto et al. reported that the 5-year OS rates after SBRT alone for stage I NSCLC including tumors <2 cm were 41%, 40%, and 52%, respectively [5-7].** Timmerman and Shibamoto reported the 5-year OS rates after SBRT alone to be 40% and 52%, respectively [6,7].

**Comment 7:** 24 out of 32 patients died due to "other disease" (page 15, line 248-9). The high rate of competing non-cancer mortality can diminish the incidence of cancer-related events, which has been an issue in comparing SBRT studies. It is essential to discuss how competing non-cancer mortality affects the assessment of treatment outcomes.

**Response 7:**We explained the 24 patients who died of other diseases with their survivals as follows:

**Line 361 – 367: During the follow-up period, 31 (48%) patients died, of which 7 died of the primary disease and 24 died of other diseases. Of the latter 24 patients, 18 patients died of non-cancer diseases, 5 died of cancer of other organs, and 1 died of secondary lung cancer. Although the median OS of the 7 patients who died of the primary disease was 44 (range, 24–89; IQR, 35–65) months, that of the 24 patients who died of other diseases was 60 (range, 3–96; IQR, 33–73) months.**

**Line 463 – 465: The median OS in the 24 patients who died of other diseases was 60 months, which did not make a large impact on the 74-month median OS of the entire patients.**

**Comment 8:** In the conclusion, it is inappropriate to suggest that combined treatment improved survival, as this is a single-arm cohort study without a comparison group. Furthermore, stating that the rates of adverse events were acceptable significantly understates the 38% incidence of pneumothorax requiring chest drainage and possibly hospitalisation. This incidence does not compare favorably with the low occurrence of adverse events observed following SBRT alone. Conclusions should take into account the significant limitations of the study.

**Response 8:** We discussed about the pneumothorax after cryoablation and the limitation of the study as follows:

**Line 500 – 504: Pneumothorax, a known complication of cryoablation or RFA, occurred in 40% of patients in the present study.** Pneumothorax is known to be associated with cryoablation or RFA, of its incidence in the present study being 38%. However, the median drainage period was only 2 days, which was not a severe event.

**Line 505 – 519**:

This study has the following limitations: (1) the retrospective descriptive design **with only 64 patients** and the lack of a control group impeded establishment of a causal inference between the treatment and outcome; and (2) a biopsy of the primary tumor was not routinely conducted at the time of distant metastases; therefore, LC could not be precisely estimated.

**Conclusion**

**The SBRT-cryoablation treatment is a feasible treatment for stage I NSCLC ≥2 cm. Because the study was a single-arm cohort study, it cannot be compared with the studies of SBRT alone, all of which were also single-arm ones. This study may contribute to increasing treatment options in patients with stage I NSCLC ≥2 cm.** SBRT followed by cryoablation is a feasible treatment option for T1-2N0M0 non-small cell lung cancer with tumor size ≥2 cm, with improved survival and acceptable rates of adverse events, compared to stereotactic body radiation alone.

**Comment 9:** Comparing Kaplan-Meier graphs for OS and DSS (Fig. 4 and 6), the number of patients at risk is nearly identical in both graphs. Given that 75% of the deaths were due to non-cancer causes, there should be a notable difference in the numbers at risk. The statistical analyses were performed using Excel, which I understand does not have a built-in Kaplan-Meier graph function and thus requires a third-party add-in or some manual steps. I recommend verifying the results using alternative statistical software.

**Response 9:**

In the DSS, the death caused by non-primary disease was censored, resulting in that the number at risk became same as that of OS. We did not explain it in the text.

**Comment 10:** T1-2 tumour with tumour size > 2cm as per 8th edition will be T1c-T2. I suggest using T1c-T2 instead of a lengthier description.

**Response 10:** We limited the study case as Stage I NSCLC with tumor size ≥2 cm, i.e., excluding 2 patients with T2b, as showing in Table 1. As the result, the number of cases were change from 66 to 64 patients. The text was changed as follows:

**Line 187 – 189:** In the eligibility: 1) **stage I** clinical T1-2N0M0 peripheral-type NSCLC with tumor size ≥2 cm based on the 8^th^ edition of TNM classification by the International Association for the Study of Lung Cancer [**17** 16];

**Line 327 – 339: Tumor size shows the maximum size including ground glass opacity area, of which tumor stage was T1mi or T1a in 3 patients, T1b in 17, T1c in 32, and T2a in 12. FDG-PET was performed in 61 of the 64 patients (95%).**

Between 2015 and 2023, 333 patients with cT1-2N0M0 non-small cell lung cancer with tumor size ≥2 cm underwent local treatments, including surgery, SBRT, and cryoablation (**Fig. 2**). Among 80 patients who underwent cryoablation, 66 patients, including 2 patients with T2bN0M0 (Stage II), were treated by SBRT followed by cryoablation (**Table 1**). The reasons for selecting the study treatment included Charlson comorbidity index ≥3 (n=26), FEV_1_/FVC < 70% (n=36), and age ≥ 80 years (n=23). On the other hand, 40 (61%) patients requested the study treatment rather than surgery despite having a comorbidity index <3. The FDG-PET was conducted for 62 of the 66 patients (94%).

**Comment 11:** Abstract: The last line in the Methods (page 3, line 50), “Median follow-up duration was 73 months”, is a repetition since the median follow-up is mentioned in the first paragraph

**Response 11:**

**Line 51-52:** In the objectives of abstract, I deleted the median follow-up duration was 73 months as follows: with 73 months of median follow-up.

**Comment 12:** Table 1: The breakdown of T stage % adds up to only 91%.

**Response 12:**

As a result of changing the number of cases from 66 to 64, the Table 1 was changed also. Thank you for your indicating my mistake.

**Line 679 – 705**:

Table 1. Patient characteristics

Characteristics	Value

Number of patients	**64** 66 (100%)

Age (y)	76 ± 7 (range: 58 – 89)

Sex	Male	**41** 42 (64)

Female	**23** 24 (36)

FEV_1_/FVC (%)	**66** 67 ± 13 (range: 33 - 89)

%FEV_1_	**96 ± 30** 98 ± 31 (range: 38 - 184)

Charlson comorbidity index	**2.2** 2.1 ± 1.3 (range: 0 - 7)

Tumor histology

Adenocarcinoma	**41** 43 (**64** 65)

Squamous cell carcinoma	23 (**36** 35)

Tumor size (cm)	2.7 ± **0.5** 0.7 (range: 2.0 – **4.0** 5.4)

2 － 3	48 (**75** 73)

>3 － 4	**16** 15 (**25** 23)

>4 － 5	2 (3)

>5 － 6	1 (2)

T-stage

T1mi or T1a	　3 (5)

T1b	17 (18)

T1c	**32** 33 (**50** 48)

T2a	**12** 11 (**18** 17)

T2b	2 (3)

SUV on PET	**4.9 ± 4.2** 5.0 ± 4.1 (range: 0.6 – 17)

FEV_1_/FVC: Forced expiratory volume in 1 second / forced vital capacity.

**SUV, standardized uptake value; PET, positron emission tomography**

**Comment 13:** Tables 3 and 4: the last column representing the cause of mortality is labelled “Prognosis, " which refers to the chance of an event happening. I suggest labelling it mortality and mentioning whether it is due to cancer or other causes for each patient.

**Response 13:** I explained as follows:

**Line 361 – 367: During the follow-up period, 31 (48%) patients died, of which 7 died of the primary disease and 24 died of other diseases. Of the latter 24 patients, 18 patients died of non-cancer diseases, 5 died of cancer of other organs, and 1 died of secondary lung cancer. Although the median OS of the 7 patients who died of the primary disease was 44 (range, 24–89; IQR, 35–65) months, that of the 24 patients who died of other diseases was 60 (range, 3–96; IQR, 33–73) months.**

**Line 745 -780:** I changed from “dead with the disease” to “dead with the primary disease”.

**Supplement Table 1** Table 3. Characteristics of patients who presented local recurrence

No. Age/Sex Tumor size Histology Local recurrence Treatment	Prognosis (months)

1	70/F	3.1 cm	Sq	61 months	None	Dead with other disease (61)*

2	86/M	2.3 cm	Ad	33 months	None	Dead with **primary** disease (41)

3	68/M	2.8 cm	Sq	16 months	Surgery	Dead with **primary** disease (44)

4	58/M	2.6 cm	Ad	13 months	Chemo	Dead with **primary** disease (29)

5	78/M	3.0 cm	Sq	8 months	Surgery	Alive without disease (9)

M: male; F: female; Ad: adenocarcinoma; Sq: squamous cell carcinoma,

*: Local recurrence found at autopsy for other disease.

**Supplement Table 2. Characteristics of patients who suffered pneumonitis**

**Pneumonitis**

**No. Age/Sex	FEV_1_/FVC(%) Tumor size Onset	Grade	Prognosis (months)**

**1	80/F	79	3.1 cm	7 weeks	2	Dead with other disease (87)**

**2	79/F	55	2.2 cm	9 weeks	3	Dead with other disease (61)**

**3	82/F	71	2.7 cm	11 weeks	2	Dead with other disease (61)**

**4	80/M	58	4.0 cm	12 weeks	2	Dead with primary disease (57)**

**5	77/F	71	2.4 cm	15 weeks	2	Alive without disease (72)**

**FEV_1_/FVC, forced expiratory volume in 1 second / forced vital capacity;**

**Onset, onset time of pneumonitis after stereotactic radiation therapy;**

**M, male; F, female.**

Table 4. Characteristics of patients who suffered pneumonia

Pneumonia

No. Age/Sex	Tumor size Type	Onset	Grade	Prognosis (months)

1	80/F	3.1 cm	BP	7 weeks	2	Dead with other disease (87)

2	79/F	2.2 cm	RP	9 weeks	3	Dead with other disease (61)

3	82/F	2.7 cm	RP	11 weeks	2	Dead with other disease (61)

4	80/M	4.2 cm	RP	12 weeks	2	Dead with disease (57)

5	82/F	5.0 cm	RP	13 weeks	2	Dead with disease (73)

6	77/F	2.4 cm	RP	15 weeks	2	Alive without disease (72)

Onset: onset time of pneumonia after stereotactic radiation therapy,

M: male; F: female; BP: broncho-pneumonia; RP: radiation pneumonitis.

**Comment 14:** The terms pneumonia and pneumonitis are used interchangeably to describe radiation pneumonitis. I suggest using radiation pneumonitis for consistency. This applies to tables 4 and 7 as well.

**Response 14:** As you say, because the pneumonia was the radiation pneumonitis, we defined the pneumonia in 5 patients as radiation pneumonitis as follows:

**Line 392 – 399:** **Five (8%) patients developed radiation pneumonitis (CTCAE grade 2 in 4 patients and grade 3 in 1 patient) between 7 and 15 weeks after treatment (Supplement Table 2).** Six (9%) patients developed pneumonitis with grade 2 (n=5) and grade 3 (n=1) on CTCAE criteria between 7 and 15 weeks after the treatment (**Table 4**); among them, five had radiation pneumonitis and one had bronchopneumonia. A**ll these patients** All of them recovered without any sequelae and survived between 57 and 87 months after the treatment.

**Line 759 – 783:** In the Supplement Table 2, we wrote it as pneumonitis.

**Supplement Table 2. Characteristics of patients who suffered pneumonitis**

**Pneumonitis**

**No. Age/Sex	FEV_1_/FVC(%) Tumor size Onset	Grade	Prognosis (months)**

**1	80/F	79	3.1 cm	7 weeks	2	Dead with other disease (87)**

**2	79/F	55	2.2 cm	9 weeks	3	Dead with other disease (61)**

**3	82/F	71	2.7 cm	11 weeks	2	Dead with other disease (61)**

**4	80/M	58	4.0 cm	12 weeks	2	Dead with primary disease (57)**

**5	77/F	71	2.4 cm	15 weeks	2	Alive without disease (72)**

**FEV_1_/FVC, forced expiratory volume in 1 second / forced vital capacity;**

**Onset, onset time of pneumonitis after stereotactic radiation therapy;**

**M, male; F, female.**

Table 4. Characteristics of patients who suffered pneumonia

Pneumonia

No. Age/Sex	Tumor size Type	Onset	Grade	Prognosis (months)

1	80/F	3.1 cm	BP	7 weeks	2	Dead with other disease (87)

2	79/F	2.2 cm	RP	9 weeks	3	Dead with other disease (61)

3	82/F	2.7 cm	RP	11 weeks	2	Dead with other disease (61)

4	80/M	4.2 cm	RP	12 weeks	2	Dead with disease (57)

5	82/F	5.0 cm	RP	13 weeks	2	Dead with disease (73)

6	77/F	2.4 cm	RP	15 weeks	2	Alive without disease (72)

Onset: onset time of pneumonia after stereotactic radiation therapy,

M: male; F: female; BP: broncho-pneumonia; RP: radiation pneumonitis.

**Comment 15:** I suggest labelling Fig. 2 as a Consort flow diagram instead of “ patient algorithm”. Use terms such as assessed for eligibility, excluded, included in analysis, etc, to label each box clearly.

**Response 15:**

**In a new Figure 3:** “Patient algorithm” was changed to “Consort flow diagram”, as you indicated, with showing “excluded” and “included in analysis”.

**Comment 16:** I suggest labelling the last subheading as Discussion rather than Comments.

**Response 16:** We changed it as follows:

**Line 431:** **Discussion Comment**

**Comment 17:** here is use of the term “other disease” in several places, which I presume refers to a disease other than cancer. However, I am unsure if it excludes second cancers. This needs clarification.

**Response 17:**

**Line 361 – 364:** I clarified it as follows: **During the follow-up period, 31 (48%) patients died, of which 7 died of the primary disease and 24 died of other diseases. Of the latter 24 patients, 18 patients died of non-cancer diseases, 5 died of cancer of other organs, and 1 died of secondary lung cancer.**

**Response to the comment of reviewer #3:**

**Comment 1:**  Line 100-102: Sentence not clear.

**Response 1:** W rewrote it as follows:

**Line 124 – 129:** **The RTOG 0618 trial reported a 4-year OS rate of 56% for SBRT in 26 patients with T1–T2 N0M0 NSCLC [5]. The RTOG 0236 study reported a 5-year OS rate of 40% in 55 patients with inoperable stage I NSCLC [6].** The study conducted by Timmerman et al. on SBRT for stage I NSCLC showed 56% of 3-year study [2], which was decreased to 40% in their 5-year assessment [6].

**Comment 2:** Line 131-136: I don’t see any information on setting.

**Response 2:** We deleted “setting” as follows

**Line 176: Study design and setting**

**Comment 3:** Line 135: Are those numbers in the brackets dates or what?

**Response 3:**We explained it as follows:

**Line 179 – 181:** The study was approved in 2015 (**approval no.,**15-011) and 2020 (**approval no.,** 20-03) by the two institutional ethics committees **of the two participating institutions.**

**Comment 4:** Line 146: What cut off was classified as old age?

**Response 4:**We explained the cut off of age as follows:

**Line 190 – 192:** (3) patients ineligible for surgery **due to comorbidities or low forced expiratory volume in 1 s (FEV_1_) or those ≥80 years old** due to comorbidities, low FEV1, or old age

**Comment 5:** Line 148-149: Did you have to test before determining if tumor was outside the reach of the probe?

**Response 5:** We explained “outside the reach of the cryoprobe” as follows:

**Line 195 – 198:** and (2) tumors located outside the reach of the cryoprobe tumors located outside the reach of the cryoprobe, **such as the location nearby hilar pulmonary artery or vein, aorta, superior vena cava, heart, and esophagus.**

**Comment 6:** Line 222: P≤0.15?? Why?

**Response 6:**We changed it from P≤0.15 to P<0.05 as follows:

**Line 308 – 309:** Variables with **P < 0.05** P≤0.15 in the univariate analysis were entered into the multivariate analysis.

**Comment 7:** Line 233-236: Were there multiple reasons for participation? Numbers seem to exceed 66 by a lot.

**Response 7:** I rewrote it as follows:

**Line 323 – 339:** **The reasons for selecting the SBRT-cryoablation treatment included a Charlson comorbidity index ≥3 or FEV_1_/FVC <70% or age ≥80 years in 28 patients (44%); 36 (56%) patients requested the SBRT-cryoablation treatment rather than surgery without those unfavorable factors.**

Between 2015 and 2023, 333 patients with cT1-2N0M0 non-small cell lung cancer with tumor size ≥2 cm underwent local treatments, including surgery, SBRT, and cryoablation (**Fig. 2**). Among 80 patients who underwent cryoablation, 66 patients, including 2 patients with T2bN0M0 (Stage II), were treated by SBRT followed by cryoablation (**Table 1**). The reasons for selecting the study treatment included Charlson comorbidity index ≥3 (n=26), FEV_1_/FVC < 70% (n=36), and age ≥ 80 years (n=23). On the other hand, 40 (61%) patients requested the study treatment rather than surgery despite having a comorbidity index <3. The FDG-PET was conducted for 62 of the 66 patients (94%).

**Comment 8:** Line 239 and 241: IQR should be checked.

**Response 8:**

 I have checked the IQR (Interquartile range) values, all of which are right.

**Comment 9:** Line 243: You followed some participants for 3 months. What happened if they were not lost to follow-up? Were they deceased?

**Response 9:** We explained the patient who died of other disease as follows:

**Line 348 – 350: One patient died 3 months after the treatment due to liver cirrhosis, which was not caused by the treatment.**

**Comment 10:** Line 268: Is it the traditional p-value? Seems unusually large. Any justification for that? Line 269: You set p value at 0.05, so why do you consider 0.09 significant.

**Response 10:** We explained it as follows:

**Line 308 – 310:** Variables with **P < 0.05** P≤0.15 in the univariate analysis were entered into the multivariate analysis.

**Line 313:** Statistical significance was set at P<0.05.

**Comment 11:** Line 282: What do you mean by comment?
You could explore the influence of patient’s prognosis on the outcomes. Poor prognosis can most likely after survival.

**Response 11:** We have changed the “Comment” to Discussion as follows:

**Line 431:** **Discussion Comment**

**Comment 12:** You could explore the influence of patient’s prognosis on the outcomes. Poor prognosis can most likely after survival.

**Response 12:** To show the prognosis sufficiently, I have demonstrated overall survival, recurrence-free survival, and disease specific survival with median follow-up of 74 months, in the text and figures. There is no other way to show the prognosis. While I have not examined QOL after the treatment, I have shown the change of pulmonary function after the treatment instead, of which mean value was just 95%.

**Line 422-429:** Pulmonary function tests were performed both before and 6 months after treatment **in 59 of 64 patients (92%).** in 61 (92%) 66 patients. **A significant decrease in FEV_1_ was observed between pretreatment and posttreatment (1.8 ± 0.6 vs. 1.7 ± 0.5 L, P < 0.001), with a mean post-/pre-FEV_1_ percentage of 95 ± 10% (median, 96; IQR, 90–100) (Supplement Fig. 1).** There was a significant decrease in FEV_1_ between pretreatment and 6-month posttreatment (1.8 ± 0.6 vs. 1.7 ± 0.6 L, p<0.001), of which the mean post- /pre-FEV_1_ percentage was 96 ± 9% (median, 97; IQR, 92–102) (**Fig. 7**).

**Comment 13:** Line 320-324: Don’t just state the limitations of the studies but proceed to provide suggestions for future steps.
Work on conclusion and make it better.

**Response 13:** We added the following:

**Line 515 – 516:** **This study may contribute to increasing treatment options in patients with stage I NSCLC ≥2 cm.**

**Response to the comment of reviewer #4:**

**Comment 1:** However, despite the clinical relevance and the encouraging outcomes, the lack of a control group, unclear patient selection criteria, and potential selection bias make it difficult to draw strong conclusions about the superiority or added benefit of the combined approach. The conclusions, as currently written, overstate the findings beyond what the data can support.

**Response 1:** In the conclusion, I rewrote as follows:

**Line 512 – 519**: **The SBRT-cryoablation treatment is a feasible treatment for stage I NSCLC ≥2 cm. Because the study was a single-arm cohort study, it cannot be compared with the studies of SBRT alone, all of which were also single-arm ones. This study may contribute to increasing treatment options in patients with stage I NSCLC ≥2 cm.** SBRT followed by cryoablation is a feasible treatment option for T1-2N0M0 non-small cell lung cancer with tumor size ≥2 cm, with improved survival and acceptable rates of adverse events, compared to stereotactic body radiation alone.

**Comment 2:** Justification for SBRT and Cryoablation Strategy
The rationale behind applying cryoablation after SBRT is not adequately justified. Authors suggest that OS might improve by preventing distant recurrence or eradicating residual tumor, but this hypothesis requires more mechanistic and clinical justification

**Response 2:** We have written a rationale as follows:

**Line 106 – 121:** Stereotactic body radiotherapy (SBRT) **is the first choice of treatment** has become the first choice of treatment for inoperable patients with clinical stage I non-small cell lung cancer (NSCLC) [1-7]. **However, previous studies have shown a significant lower rate of overall survival (OS) than that of local control (LC) (40–83% vs. ≥90%) [1-8].** However, there are significant differences between the local control (LC) and overall survival (OS) rates in these reports (≥90% vs. 43–83% respectively). In addition, a **A meta-analysis reported a significantly shorter OS following SBRT compared to surgery** A meta-analysis found that surgery led to a significantly longer OS than SBRT in patients with stage I NSCLC [**9** 8]. Although the **short** low OS rate after SBRT could be attributed to a high comorbidity in inoperable patients, it could also result from residual tumors within **large scars** scar tissue that developed after SBRT. **These residual tumors are usually difficult to detect on computed tomography (CT) and may metastasize, which would decrease OS after SBRT.** , which are usually hard to detect on computed tomography (CT) and could metastasize, and thus would decrease the OS after SBRT.

**Line 139 – 150:** We have added a rationale as follows: However, the LC rates after both cryoablation and RFA are **reportedly** lower for NSCLCs with tumor size ≥2 cm than those <2 cm [**11,12** 10,11], which is consistent with the findings regarding SBRT **results** [9]. **The “2cm” tumor size is associated with some limitations for ablation therapies and SBRT.**

**The mechanism of tumor control differs for SBRT, cryoablation, and RFA. SBRT causes injury to the tumor DNA. Cryoablation induces the formation of ice crystals, leading to cell membrane destruction and subsequent intracellular dehydration. RFA leads to coagulation necrosis due to high temperatures. Owing to the different mechanisms, a combination of these therapies may improve LC for stage I NSCLC with tumor size ≥2 cm; however, this combination therapy has not been explored.**

**Line 452 - 468**: We have added a rationale as follows: **With the SBRT-cryoablation treatment, the 3-year OS rate was 87%,** In this study, the 3-year OS rates was 87%, which **was** is equal to or higher than that **of previous** reported in studies on SBRT alone (43%–83%) [1-**4** 5]. However, **the** a 3-year duration is too short to estimate **the** OS of patients with stage I NSCLC, which should be ≥5 years. **The RTOG 0915 and RTOG 0236 studies and Shibamoto et al. reported that the 5-year OS rates after SBRT alone for stage I NSCLC including tumors <2 cm were 41%, 40%, and 52%, respectively [5-7].** Timmerman and Shibamoto reported the 5-year OS rates after SBRT alone to be 40% and 52%, respectively [6,7]. The 75% of the 5-year OS rate in our study for tumors ≥2 cm was higher than that in those reports, suggesting that the SBRT-cryoablation treatment can cure primary tumors and prevent distant recurrences more effectively than SBRT alone. **The median OS in the 24 patients who died of other diseases was 60 months, which did not make a large impact on the 74-month median OS of the entire patients.** Contrastingly, the 5-year OS rate in our study was 75%, which suggests that SBRT followed by cryoablation could control primary tumors and prevent distant recurrences compared to SBRT alone.

**Comment 3:** It is recommended that the Introduction and Discussion sections provide a more detailed and logically structured explanation of why this combination strategy was pursued. Ideally, this justification should be supported by relevant preclinical or clinical literature. Including references that explain potential synergistic effects or biological rationale would strengthen the scientific foundation of the study design.

**Response 3:** Introduction was rewritten as follows:

**Line 106 – 173:** Stereotactic body radiotherapy (SBRT) **is the first choice of treatment** has become the first choice of treatment for inoperable patients with clinical stage I non-small cell lung cancer (NSCLC) [1-7]. **However, previous studies have shown a significant lower rate of overall survival (OS) than that of local control (LC) (40–83% vs. ≥90%) [1-8].** However, there are significant differences between the local control (LC) and overall survival (OS) rates in these reports (≥90% vs. 43–83% respectively). In addition, a **A meta-analysis reported a significantly shorter OS following SBRT compared to surgery** A meta-analysis found that surgery led to a significantly longer OS than SBRT in patients with stage I NSCLC [**9** 8]. Although the **short** low OS rate after SBRT could be attributed to a high comorbidity in inoperable patients, it could also result from residual tumors within **large scars** scar tissue that developed after SBRT. **These residual tumors are usually difficult to detect on computed tomography (CT) and may metastasize, which would decrease OS after SBRT.** , which are usually hard to detect on computed tomography (CT) and could metastasize, and thus would decrease the OS after SBRT. Additionally, most studies on SBRT **have** reported **a** the 3-year OS rate [1-**4** 5], which is too short **to assess** for assessing treatment outcomes in patients with stage I NSCLC. **The RTOG 0618 trial reported a 4-year OS rate of 56% for SBRT in 26 patients with T1–T2 N0M0 NSCLC [5]. The RTOG 0236 study reported a 5-year OS rate of 40% in 55 patients with inoperable stage I NSCLC [6].** The study conducted by Timmerman et al. on SBRT for stage I NSCLC showed 56% of 3-year study [2], which was decreased to 40% in their 5-year assessment [6]. Shintani et al. reported that local recurrence occurred even at >5 years after SBRT **in 7 of 216 (3%) patients with NSCLC** [**10** 9]. **These reports suggested the shortage of 3 years for the assessment of LC and OS rates after SBRT.**

**Ablation therapies, such as cryoablation or radiofrequency ablation (RFA), are** On the other hand, ablation therapies such as cryoablation or radiofrequency ablation (RFA) have been also reported as alternative therapies for stage I NSCLC [**11-14** 10-13]. The 3-year LC and OS rates after cryoablation for stage I NSCLC have been reported to be 90% and 95%, respectively, with no significant difference unlike in SBRT [**11** 10]. However, the LC rates after both cryoablation and RFA are **reportedly** lower for NSCLCs with tumor size ≥2 cm than those <2 cm [**11,12** 10,11], which is consistent with the findings regarding SBRT **results** [9]. **The “2cm” tumor size is associated with some limitations for ablation therapies and SBRT.**

**The mechanism of tumor control differs for SBRT, cryoablation, and RFA. SBRT causes injury to the tumor DNA. Cryoablation induces the formation of ice crystals, leading to cell membrane destruction and subsequent intracellular dehydration. RFA leads to coagulation necrosis due to high temperatures. Owing to the different mechanisms, a combination of these therapies may improve LC for stage I NSCLC with tumor size ≥2 cm; however, this combination therapy has not been explored.**

Combination therapy with SBRT, cryoablation, and RFA for stage I NSCLC with tumor size ≥2 cm may allow improving LC and survival; however, this remains to be studied. Although RFA reportedly increases the incidence of **pneumonitis** pneumonia in patients with NSCLC who have previously undergone external beam radiotherapy [**13** 12], cryoablation shows no difference in the incidence of adverse events between patients with and without interstitial **pneumonitis** pneumonia [**14** 13]. Therefore, cryoablation is more acceptable than RFA for combination with SBRT.

**Regarding the combination therapy of SBRT and cryoablation, SBRT should be performed first, because cryoablation decreases tumor vascularity, which in turn decreases tumor radiosensitivity [15].** In combination therapy with SBRT and cryoablation, SBRT should be performed first, because tumor radiosensitivity is dependent on intracellular oxygen concentration [14]. Because cryoablation decreases tumor vascularity, this would decrease tumor radiosensitivity. Additionally, scarring around the tumor usually occurs immediately after cryoablation, which impedes SBRT planning. In contrast, **SBRT usually does not induce inflammation** SBRT usually does not make the scar within 1 month. **In this study, cryoablation was performed 2 – 3 weeks after SBRT on 64 patients with stage I NSCLC with tumor size ≥2 cm,** Therefore, this study conducted the cryoablation 2 to 3 weeks after SBRT for 66 patients with cT1-2N0M0 NSCLC with tumor size ≥2 cm, and **the long-term outcomes were evaluated** the long-term outcomes with a median follow-up period of **74** 73 months.

**Comment 4:** - Patient Selection and Treatment Allocation
It is unclear how the treatment modality was determined among 333 patients. What were the specific indications for: Surgery, SBRT alone, Cryoablation alone, or SBRT followed by cryoablation. This ambiguity severely limits the interpretability of the study results and introduces significant selection bias. For example, cryoablation could only be performed for “tumors located within the reach of the cryoprobe,” suggesting technical feasibility was a selection factor. This should be explicitly acknowledged and addressed as a source of bias.

**Response 4:** The inclusion criteria were rewritten as follows:

**Line 186 – 203**: (1) **stage I** clinical T1-2N0M0 peripheral-type NSCLC with tumor size ≥2 cm based on the 8^th^ edition of TNM classification by the International Association for the Study of Lung Cancer [**17** 16]; (2) Eastern Cooperative Oncology Group performance status 0–2; (3) patients ineligible for surgery **due to comorbidities or low forced expiratory volume in 1 s (FEV_1_) or those ≥80 years old** due to comorbidities, low FEV1, or old age ; and (4) patients who preferred **the SBRT-cryoablation treatment** the study treatment to surgery. The exclusion criteria were as follows: (1) active interstitial **pneumonitis** pneumonia or previous treatment for interstitial **pneumonitis** pneumonia; and (2) tumors located outside the reach of the cryoprobe tumors located outside the reach of the cryoprobe, **such as the location nearby hilar pulmonary artery or vein, aorta, superior vena cava, heart, and esophagus.** **All patients received the explanation that the standard therapy for stage I NSCLC was surgery and not ablation therapies or SBRT, as these have lower LC rates compared to surgery.** When they selected SBRT followed by cryoablation, **they** all patients provided written informed consent after receiving **an** extensive explanation regarding the risks and benefits of the treatment.

**Line 318 -339:** I rewrote the results as follows: **Between 2015 and 2023, 220 patients with stage I NSCLC with tumor size ≥2 cm underwent local treatments, including surgery, SBRT, and cryoablation (Fig. 3). Of the 220 patients, 122 and 20 were treated by surgery and SBRT alone, respectively. Among the 78 patients who underwent cryoablation, 14 underwent cryoablation alone, whereas 64 received SBRT-cryoablation treatment (Table 1). The reasons for selecting the SBRT-cryoablation treatment included a Charlson comorbidity index ≥3 or FEV_1_/FVC <70% or age ≥80 years in 28 patients (44%); 36 (56%) patients requested the SBRT-cryoablation treatment rather than surgery without those unfavorable factors. Tumor size shows the maximum size including ground glass opacity area, of which tumor stage was T1mi or T1a in 3 patients, T1b in 17, T1c in 32, and T2a in 12. FDG-PET was performed in 61 of the 64 patients (95%).**

Between 2015 and 2023, 333 patients with cT1-2N0M0 non-small cell lung cancer with tumor size ≥2 cm underwent local treatments, including surgery, SBRT, and cryoablation (**Fig. 2**). Among 80 patients who underwent cryoablation, 66 patients, including 2 patients with T2bN0M0 (Stage II), were treated by SBRT followed by cryoablation (**Table 1**). The reasons for selecting the study treatment included Charlson comorbidity index ≥3 (n=26), FEV_1_/FVC < 70% (n=36), and age ≥ 80 years (n=23). On the other hand, 40 (61%) patients requested the study treatment rather than surgery despite having a comorbidity index <3. The FDG-PET was conducted for 62 of the 66 patients (94%).

**Comment 5:** - Need for Comparator Group
The study would benefit greatly from a matched SBRT-alone cohort to determine whether the additional cryoablation offers real benefit in terms of OS, LC, or adverse events. Without such a comparison, the claim that combination therapy is superior to SBRT alone is not supportable

**Response 5:**

Propensity score matching between SBRT-alone and SBRT followed by cryoablation is not possible due to few cases. We changed the discussion and conclusion as follows:

**Line 505 – 519**: This study has the following limitations: (1) the retrospective descriptive design **with only 64 patients** and the lack of a control group impeded establishment of a causal inference between the treatment and outcome; and (2) a biopsy of the primary tumor was not routinely conducted at the time of distant metastases; therefore, LC could not be precisely estimated.

**Conclusion**

**The SBRT-cryoablation treatment is a feasible treatment for stage I NSCLC ≥2 cm. Because the study was a single-arm cohort study, it cannot be compared with the studies of SBRT alone, all of which were also single-arm ones. This study may contribute to increasing treatment options in patients with stage I NSCLC ≥2 cm.** SBRT followed by cryoablation is a feasible treatment option for T1-2N0M0 non-small cell lung cancer with tumor size ≥2 cm, with improved survival and acceptable rates of adverse events, compared to stereotactic body radiation alone.

**Comment 6:** A statement like “SBRT followed by cryoablation improves survival compared to SBRT alone” (e.g., lines 60–63, 328–330) is speculative and not supported by direct comparison in this study. The study lacks a control group of patients receiving SBRT alone. Thus, the conclusion should be softened (e.g., “SBRT followed by cryoablation showed favorable outcomes…”).

**Response 6:** The conclusion in the abstract was changed as follows:

**Line 72 – 76: Conclusion:** Stereotactic body radiation therapy followed by cryoablation is a **feasible and favorable treatment for stage I non-small cell lung cancer with a tumor size ≥2 cm.** for T1-2N0M0 non-small cell lung cancer with tumor size ≥2 cm, with improved survival and acceptable rates of adverse events, compared to stereotactic body radiation alone.

**Line 512 – 519**: We changed the last conclusion as follows: **The SBRT-cryoablation treatment is a feasible treatment for stage I NSCLC ≥2 cm. Because the study was a single-arm cohort study, it cannot be compared with the studies of SBRT alone, all of which were also single-arm ones. This study may contribute to increasing treatment options in patients with stage I NSCLC ≥2 cm.** SBRT followed by cryoablation is a feasible treatment option for T1-2N0M0 non-small cell lung cancer with tumor size ≥2 cm, with improved survival and acceptable rates of adverse events, compared to stereotactic body radiation alone.

**Comment 7:** - Patterns of Recurrence
It would significantly strengthen the paper to report patterns of recurrence (local, regional, or distant) and provide detail on subsequent treatments received, especially in recurrent cases.

**Response 7:** We wrote the recurrence pattern as follows:

**Line 346 －348:** **Although 15 patients relapsed, including recurrence at local alone in 2, distant alone in 10, and both in 3, 49 patients did not experience recurrence.**

**Supplement Table 1**: The treatment methods for local recurrence were demonstrated.

**Comment 8:** - Toxicity Assessment
The distinction between bronchopneumonia (BP) and radiation pneumonitis (RP) is not clearly explained. What were the diagnostic criteria used to differentiate the two?
CTCAE version 4.03 is stated to be used in the main text, but Reference 17 refers to CTCAE v3.0, which should be corrected.

**Response 8:** The version of CTCAE was changed as follows:

**Line 270 – 271:** Posttreatment adverse events were graded based on the Common Terminology Criteria for Adverse Events (CTCAE version **3.0** 4.03) [**18** 17].

**Comment 9:** - Overuse of Figures and Tables
Consider combining Figures 3–6 into a single multipanel survival figure (e.g., Kaplan-Meier curves). Tables 3–5 may be more appropriate as Supplementary Tables, as they do not add substantial value to the main narrative.

**Response 9:** We put the Figures 3-6 together as Figure 4 as follows:

**Line 350 – 386:**

**Kaplan-Meier curves of LC, OS, RFS, and DSS are shown in Fig. 4.**

**The 3- and 5-year LC rates were both 93% (95% confidence interval [CI], 87–100%) (Fig. 4a). The median LC was 73 (range, 3–110; IQR, 36–87) months. Local recurrence was diagnosed in 5 (8%) patients, with a median LC of 16 (range, 8–61; IQR, 13–34) months (Supplement Table 1).**

Both the 3- and 5-year LC rates were 93% (95% confidence interval [CI], 87%–100%) (**Fig. 3**). Local recurrence was diagnosed in 5 (8%) patients between 8 and 61 months after the treatment (**Table 3**).

**The 3- and 5-year OS rates were 87% (CI, 78–95%) and 74% (CI, 63–86%), respectively (Fig. 4b). The median OS was 74 (range, 3–110; IQR, 41–89) months. During the follow-up period, 31 (48%) patients died, of which 7 died of the primary disease and 24 died of other diseases. Of the latter 24 patients, 18 patients died of non-cancer diseases, 5 died of cancer of other organs, and 1 died of secondary lung cancer. Although the median OS of the 7 patients who died of the primary disease was 44 (range, 24–89; IQR, 35–65) months, that of the 24 patients who died of other diseases was 60 (range, 3–96; IQR, 33–73) months.**

The 3- and 5-year OS rates were 87% (CI, 79%–96%) and 75% (CI, 64%–86%), respectively (**Fig. 4)**. During the follow-up period, 32 (48%) patients were dead, with 24 of them dying of other diseases.

**The 3- and 5-year RFS rates were 74% (CI, 63–85%) and 60% (CI, 48–73%), respectively (Fig. 4c). The median RFS was 64 (range, 3–110; IQR, 32–82) months. The median RFS of the 15 relapsed patients was 29 (range, 3–96; IQR, 33–73) months, whereas that of the 49 non-relapsed patients was 75 (range, 3–110; IQR, 44–89) months.**

The 3- and 5-year RFS rates were 74% (CI, 63%–84%) and 60% (CI, 48%–73%), respectively (**Fig. 5**). Fifteen patients (23%) experienced distant metastases between 3 and 60 months after the treatment.

**The 3- and 5-year DSS rates were 96% (CI, 92–100%) and 92% (CI, 85–100%), respectively (Fig. 4d). The median DSS was 74 (range, 3–110; IQR, 41–89) months. The median DSS of the 7 patients who died of the primary disease was 53 (range, 24–89; IQR, 38–64) months, whereas that of the 57 patients who died of other diseases was 77 (range, 3–110; IQR, 44–89) months.**

The 3- and 5-year DSS rates were 97% (CI, 92%–100%) and 93% (CI, 86%–100%), respectively (**Fig. 6)**.

**Line 657 – 669**: We put the Figures 3-6 together as Figure 4 as follows:

**Figure 4. Kaplan-Meier curves. The 95% confidence interval is depicted in the shaded area. (a) Local control. The 3- and 5-year local control rates were both 93%.; (b) Overall survival. The 3- and 5-year overall survival rates were 87% and 74%, respectively.; (c) Recurrence-free survival. The 3- and 5-year recurrence-free survival rates were 74% and 60%. respectively.; (d) Disease-specific survival. The 3- and 5-year disease-specific survival rates were 96% and 92%, respectively.**

Figure 3. Local control curve. A 95% confidence interval is shown in shade.

Figure 4. Overall survival curve. A 95% confidence interval is shown in shade.

Figure 5. Recurrence-free survival curve. A 95% confidence interval is shown in shade.

Figure 6. Disease-specific survival curve. A 95% confidence interval is shown in shade.

**Line 354 – 358:** We changed the Table 3 to Supplement Table 1 as follows: **Local recurrence was diagnosed in 5 (8%) patients, with a median LC of 16 (range, 8–61; IQR, 13–34) months (Supplement Table 1).**

Both the 3- and 5-year LC rates were 93% (95% confidence interval [CI], 87%–100%) (**Fig. 3**). Local recurrence was diagnosed in 5 (8%) patients between 8 and 61 months after the treatment (**Table 3**).

**Line 730 – 818:** We changed as follows: the Table 6 to Table 3, the Table 3 to Supplement Table 1, the Table 4 to Supplement Table 2, and the Table 5 to Supplement Table 3.

Table **3** 6. Cox proportional hazard regression analysis for overall survival

Univariate analysis	Multivariate analysis

Variables	HR (95% CI)	P	HR (95% CI)	P

Age	1.0 (1.0 － 1.1)	0.25	**―**

Sex	0.7 (0.3 － 1.5 ) 0.38	**―**

Comorbidity index	1.2 (0.9 － 1.5)	0.17	―

Tumor size	1.6 (0.8 － 3.0)	0.17	―

Tumor stage	1.7 (1.1 － 2.4) 0.02	1.8 (**1.0** 0.9 – **3.2** 2.5) 0.0**4** 9

SUV on PET	1.1 (1.0 － 1.2)	0.02	1.0 (1.0 – 1.1) **0.32** 0.29

Tumor histology (Ad vs. Sq) 1.9 (0.9 － 3.8)	0.09	―

**BED	1.0 (0.9 － 1.0)	0.02	1.0 (0.9 – 1.0)	0.02**

HR: hazard ratio; CI: confidence interval,

SUV: standard uptake value; PET: positron emission tomography,

Ad: adenocarcinoma; Sq: squamous cell carcinoma.

**BED, biological effective dose.**

**Supplement Table 1** Table 3. Characteristics of patients who presented local recurrence

No. Age/Sex Tumor size Histology Local recurrence	Treatment	Prognosis (months)

1	70/F	3.1 cm	Sq	61 months	None	Dead with other disease (61)*

2	86/M	2.3 cm	Ad	33 months	None	Dead with **primary** disease (41)

3	68/M	2.8 cm	Sq	16 months	Surgery	Dead with **primary** disease (44)

4	58/M	2.6 cm	Ad	13 months	Chemo	Dead with **primary** disease (29)

5	78/M	3.0 cm	Sq	8 months	Surgery	Alive without disease (9)

M: male; F: female; Ad: adenocarcinoma; Sq: squamous cell carcinoma,

*: Local recurrence found at autopsy for other disease.

**Supplement Table 2. Characteristics of patients who suffered pneumonitis**

**Pneumonitis**

**No. Age/Sex	FEV_1_/FVC(%) Tumor size	Onset	Grade	Prognosis (months)**

**1	80/F	79	3.1 cm	7 weeks	2	Dead with other disease (87)**

**2	79/F	55	2.2 cm	9 weeks	3	Dead with other disease (61)**

**3	82/F	71	2.7 cm	11 weeks	2	Dead with other disease (61)**

**4	80/M	58	4.0 cm	12 weeks	2	Dead with primary disease (57)**

**5	77/F	71	2.4 cm	15 weeks	2	Alive without disease (72)**

**FEV_1_/FVC, forced expiratory volume in 1 second / forced vital capacity;**

**Onset, onset time of pneumonitis after stereotactic radiation therapy;**

**M, male; F, female.**

Table 4. Characteristics of patients who suffered pneumonia

Pneumonia

No. Age/Sex	Tumor size Type	Onset	Grade	Prognosis (months)

1	80/F	3.1 cm	BP	7 weeks	2	Dead with other disease (87)

2	79/F	2.2 cm	RP	9 weeks	3	Dead with other disease (61)

3	82/F	2.7 cm	RP	11 weeks	2	Dead with other disease (61)

4	80/M	4.2 cm	RP	12 weeks	2	Dead with disease (57)

5	82/F	5.0 cm	RP	13 weeks	2	Dead with disease (73)

6	77/F	2.4 cm	RP	15 weeks	2	Alive without disease (72)

Onset: onset time of pneumonia after stereotactic radiation therapy,

M: male; F: female; BP: broncho-pneumonia; RP: radiation pneumonitis.

**Supplement Table 3. Cox proportional hazard regression analysis**

**for local recurrence**

**Univariate analysis**

**Variables	HR (95% CI)	P-value**

**Tumor size	1.5 (0.3 － 7.0)	0.62**

**Tumor stage	2.0 (0.6 － 6.9)	0.27**

**Tumor histology (Ad vs. Sq)	3.1 (0.5 － 18.3)	0.22**

**SUV on PET	1.1 (0.9 － 1.3)	0.53**

**BED	1.0 (0.9－ 1.1)	0.63**

**HR, hazard ratio; CI, confidence interval;**

**Ad, adenocarcinoma; Sq, squamous cell carcinoma;**

**SUV, standardized uptake value; PET, positron emission tomography;**

**BED, biological effective dose.**

Table 5. Cox proportional hazard regression analysis for local recurrence

Univariate analysis

Variables	HR (95% CI)	P

Tumor size	1.0 (0.3 － 3.5)	0.95

Tumor stage	1.5 (0.6 － 4.1)	0.39

Tumor histology (Ad vs. Sq)	3.2 (0.5 － 19.3)	0.20

SUV on PET	1.1 (0.9 － 1.3)	0.57

HR: hazard ratio; CI: confidence interval,

Ad: adenocarcinoma; Sq: squamous cell carcinoma,

SUV: standardized uptake value; PET: positron emission tomography.

**Line 812 – 821:** We deleted the Table 7 as follows:

Table 7　Logistic regression analysis for posttreatment pneumonia

Univariate analysis	Multivariate analysis

Variables	OR (95% CI)	P	OR (95% CI)	P

Age	1.1 (1.0 － 1.3)	0.15	0.9 (0.8 - 1.0)	0.18

Comorbidity index	0.9 (0.5 － 1.7)	0.76	―

FEV_1_/FVC	1.0 (1.0 － 1.1)	0.59	―

Tumor size	2.1 (0.8 － 5.3)	0.11	0.6 (0.2 – 1.9)	0.36

V20	1.9 (0.8 － 4.6)	0.14	0.7 (0.3 － 2.0)	0.51

FEV1/FVC: Forced expiratory volume in 1 second / forced vital capacity.

**Line 422 – 429**: We explained its content as follows:

Pulmonary function tests were performed both before and 6 months after treatment **in 59 of 64 patients (92%).** in 61 (92%) 66 patients. **A significant decrease in FEV_1_ was observed between pretreatment and posttreatment (1.8 ± 0.6 vs. 1.7 ± 0.5 L, P < 0.001), with a mean post-/pre-FEV_1_ percentage of 95 ± 10% (median, 96; IQR, 90–100) (Supplement Fig. 1).** There was a significant decrease in FEV_1_ between pretreatment and 6-month posttreatment (1.8 ± 0.6 vs. 1.7 ± 0.6 L, p<0.001), of which the mean post- /pre-FEV_1_ percentage was 96 ± 9% (median, 97; IQR, 92–102) (**Fig. 7**).

**Comment 10:** Many references are more than a decade old. Recent data on SBRT, cryoablation, and combination therapies should be incorporated to better contextualize the study findings.

**Response 10:** We changed the references as follows:

**Line 527 – 566:**

We deleted old one i.e., Timmerman R, Paulus R, Galvin J, Michalski J, Straube W, Bradley J, et al. [Stereotactic body radiation therapy for inoperable early-stage lung cancer.](https://pubmed.ncbi.nlm.nih.gov/20233825/) JAMA. 2010 Mar 17;303(11):1070-6. doi:10.1001/jama.2010.261. PMID: 20233825.

We added new ones as follows:

**5. Timmerman RD, Paulus R, Pass HI, Gore EM, Edelman MJ, Galvin J, et al.** [**Stereotactic Body Radiation Therapy for Operable Early-Stage Lung Cancer: Findings from the NRG Oncology RTOG 0618 Trial.**](https://pubmed.ncbi.nlm.nih.gov/29852037/) **JAMA Oncol. 2018 Sep 1;4(9):1263-1266. doi:10.1001/jamaoncol. 2018.1251. PMID: 29852037.**

**8. Videtic GM, Paulus R, Singh AK, Chang JY, Parker W, Olivier KR, et al.** [**Long-term Follow-up on NRG Oncology RTOG 0915 (NCCTG N0927): A Randomized Phase 2 Study Comparing 2 Stereotactic Body Radiation Therapy Schedules for Medically Inoperable Patients with Stage I Peripheral Non-Small Cell Lung Cancer.**](https://pubmed.ncbi.nlm.nih.gov/30513377/) **Int J Radiat Oncol Biol Phys. 2019 Apr 1;103(5):1077-1084. doi: 10.1016/j.ijrobp.2018.11.051. Epub 2018 Dec 1.PMID: 30513377.**

**Response to the comment of reviewer #5:**

**Comment 1:** The manuscript presents a well-designed retrospective cohort study on the long-term outcomes of combined SBRT and cryoablation for cT1-2N0M0 NSCLC with tumor size ≥2 cm. The median follow-up of 73 months and focus on a clinically relevant population – patients ineligible or unwilling to undergo surgery – enhance the significance of this work. The results are clearly reported and show promising local control and overall survival outcomes.
However, several areas require clarification, elaboration, and improvement to strengthen the manuscript's scientific rigor and clinical impact. Please refer to the attached documents of suggested revision.

**Response 1:**

Thank you for many valuable comments including yours. I revised the manuscript as responses for other comments, all of which are written above.
