# Supplementary material for: Long-term outcomes of combination therapy with stereotactic body radiation therapy plus cryoablation using liquid nitrogen for stage I non-small cell lung cancer with tumors ≥2 cm
Source: PLoS One. 2025 Oct 8;20(10):e0332893. doi: 10.1371/journal.pone.0332893 (PMC12507226; doi:10.1371/journal.pone.0332893)
Supplement: Attachment — Submitted filename: 2025.8.31.Response to the reviewer.docx [file pone.0332893.s010.docx]

2025 Aug 31

Ms. Ref. No.: PONE-D-25-05871

Dear Editor in chief,

Long-term outcomes of combination therapy with stereotactic body radiation therapy plus cryoablation for T1-2N0M0 non-small cell lung cancer with tumor size ≥2 cm

Response to the comments of the reviewer #5.

Thank you for your advice.

I have revised the paper according to the professional editing, which is the 3^rd^ time.

If there were some problems, please let me know.

Sincerely,

Hiroaki Nomori, MD,

Department of thoracic surgery,

Kashiwa Kousei General Hospital

Chiba Japan.

E-mail: hnomori@qk9.so-net.ne.jp
